# Drivers of insect herbivory resistance and tolerance to plant damage in the *Brachypodium distachyon* species complex

**DOI:** 10.1002/ajb2.70176

**Published:** 2026-03-16

**Authors:** Antonio J. Manzaneda, Luisa M. Martínez, Ana Fernández‐Ocaña, Teresa Salido, Pedro J. Rey

**Affiliations:** ^1^ Departamento de Biología Animal, Biología Vegetal y Ecología Universidad de Jaén Jaén E‐23071 Spain; ^2^ Instituto Universitario de Investigación en Olivar y Aceites de Oliva. Campus Las Lagunillas Jaén E‐23071 Spain; ^3^ Instituto Interuniversitario de Investigación del Sistema Tierra en Andalucía (IISTA‐UJA) Campus Las Lagunillas Jaén E‐23071 Spain

**Keywords:** allopolyploidy, Brachypodium hybridum, Brachypodium stacei, C:N ratio, functional traits, insect herbivory, palatability, Poaceae, silica content, tolerance

## Abstract

**Premise:**

Insect herbivory is a major biotic factor shaping plant populations and driving the evolution of defensive traits. Polyploidy (whole‐genome duplication) often induces substantial phenotypic and genotypic changes that may affect species interactions, including herbivory. However, natural variation in herbivory responses and the drivers of resistance and tolerance across heteroploid lineages remain poorly understood.

**Methods:**

We conducted a bioassay to quantify variation in plant damage and tolerance to locust herbivory across multiple diploid and allotetraploid populations of the *Brachypodium distachyon* species complex, a model system comprising two diploid species (*B. distachyon* and *B. stacei*) and their allotetraploid derivative (*B. hybridum*). For each species, we also examined which plant functional traits were associated with resistance and tolerance to herbivory.

**Results:**

Herbivory reduced maternal fitness across the species complex, although its magnitude depended on species and the fitness component considered. Our results do not support enhanced herbivory resistance or tolerance in the allotetraploid lineage: Levels of plant damage in *B. hybridum* were comparable to those of one diploid parent (*B. distachyon*), and diploid *B. distachyon* had higher tolerance than *B. hybridum* for two of three fitness estimators. Variation in resistance was associated with differences in plant traits, particularly C:N ratio and silica content. In *B. distachyon*, tolerance was negatively associated with silica and water content, suggesting allocation trade‐offs between resistance‐ and tolerance‐related traits.

**Conclusions:**

Overall, our findings indicate that variation in herbivory responses across *Brachypodium* populations is more closely linked to population history and trait differentiation than to polyploid formation per se.

Insect herbivory is a major ecological biotic factor that impacts plant populations and productivity in both managed and natural vegetation (Strauss and Zangerl, 2002). Across all vegetation systems, foliage, sap, and root‐feeding herbivores may remove up to 20% of net plant productivity (Agrawal, 2011; Mitchell et al., 2016). Beyond its impact on productivity, herbivory is also a primary driver of plant trait selection and ecological community structuring (Becerra, 2007; Agrawal, 2011).

For plants, the amount of herbivory received depends mainly on the balance between their ability to produce defensive traits that deter herbivore feeding (i.e., resistance) and their nutritional quality (Agrawal and Fishbein, 2006). Once herbivore damage occurs, its impact on plant fitness is essentially determined by the plant's ability to regrow and reproduce after herbivory, namely, its tolerance to herbivory (Strauss and Agrawal, 1999; Fornoni, 2011). Together, resistance to herbivory, tolerance to damage, and plant nutritional status determine the plant response to herbivory (Núñez‐Farfán et al., 2007; Züst and Agrawal, 2017). This response pivots on a set of several functional traits of complex nature under polygenic control (e.g., specific leaf area, dry matter and water content, C:N ratio, concentration of secondary compounds in tissues, silica content; Agrawal and Fishbein, 2006; Agrawal, 2011; Mitchell et al., 2016). Although intra‐ and interspecific variation in plant response is dependent on the ecological context, plant response variation to herbivory hinges primarily on the natural variation and plasticity of these functional traits (Reese et al., 2016).

Among all the evolutionary processes, polyploidization (whole‐genome duplication) typically induces dramatic genotypic and phenotypic changes that may impact species interactions, including herbivory (reviewed by te Beest et al., 2012; Segraves and Anneberg, 2016). Polyploidy can influence phenotypic expression through gene‐dosage effects, hybridization, and changes in regulatory networks (Doyle and Coate, 2019). Likewise, genome‐size‐driven nucleotypic effects may also independently shape plant phenotypes (Bennett, 1987; Doyle and Coate, 2019). These effects arise from the absolute amount of nuclear DNA and can modify cell‐cycle duration, cell size, growth rates, and metabolic allocation, with potential consequences for ecophysiological traits (Bennett, 1987; Knight et al., 2005) that in turn, may be important in ecological interactions. Therefore, although herbivore differential selection may drive phenotypic differentiation between ploidies, polyploidy itself may also alter key plant traits that mediate plant–herbivory interactions through shifts in secondary defensive chemistry or nutritional traits (Hull‐Sanders et al., 2009; Edger et al., 2015; Barco and Clay, 2019; but see Gaynor et al., 2020). However, whether such trait modifications impact the herbivory pattern between different ploidy lineages is not yet clear. Thus, although enhanced antiherbivore defenses have been shown in some ploidy complexes (e.g., Hull‐Sanders et al., 2009), herbivore selection on cytotypes appears variable and species‐specific (see, e.g., Arvanitis et al., 2010; Stutz et al., 2016; Harms and Walter, 2021). Regarding tolerance, theory predicts that (1) higher vigor in polyploids caused by whole‐genome duplications and/or ancient hybridity, and (2) increased cell size typical of polyploidy and/or from nucleotypic effects that may provide a rapid regeneration of tissues (Scholes and Paige, 2014), higher‐level polyploids may show enhanced tolerance to herbivory compared to their diploid ancestors. However, empirical evidence for this hypothesis is scant and contradictory (Boalt et al., 2010; König et al., 2014; Scholes, 2020). Similarly, the specific plant traits that correlate with tolerance to damage between different ploidy lineages are not known.

Here, we comprehensively analyzed resistance and tolerance to insect damage in the *Brachypodium distachyon* (L.) P. Beauv. (Poaceae) species complex, a plant model used to address ecological, genetic, and evolutionary questions in temperate grasses (Scholthof et al., 2018). The three species that comprise this system are well characterized at the genetic, phylogenetic, and cytogenetic levels. Thus, the natural allotetraploid *Brachypodium hybridum stacei* Catalán, Joch. Müll, Hasterok & Jenkins (2*n* = 4*x* = 30) is derived from bidirectional crosses of its parents *Brachypodium distachyon* (2*n* = 2*x* = 10) and *Brachypodium stacei* Catalán, Joch. Müll, & Langdon (2*n* = 2*x* = 20), which have occurred repeatedly between ~1.4 and ~0.14 million years ago (Catalán et al., 2012; Gordon et al., 2020). Genome size also differs significantly among species: Diploids *B. distachyon* and *B. stacei* have small but distinct genomes (≈0.63 and 0.56 pg 2 C, respectively), while the allotetraploid *B. hybridum* combines approximately both subgenomes (≈1.26 pg 2 C) (Catalán et al., 2012). In this complex, *B. stacei* is the oldest lineage, arising ~10 million years ago, while *B. distachyon* emerged ~7 million years ago (Scholthof et al., 2018). Across its native distribution range, the three species are ecologically differentiated; *B. stacei* and *B. hybridum* are found in drier locations than *B. distachyon* diploids (Manzaneda et al., 2012; López‐Alvarez et al., 2015). Such ecological differentiation encompasses interspecific and interpopulational variation in functional traits (Martínez et al., 2018), which may directly or indirectly influence insect herbivory (Agrawal, 2011; Hall et al., 2020). We showed previously that *B. hybridum* polyploids have higher specific leaf area (SLA) and water content (WC) than in *B. distachyon* but similar to those in *B. stacei* (Martínez et al., 2018). Similarly, silicon has the potential for deterring insect herbivory in *B. distachyon* (Hall et al., 2020; Biru et al., 2021). However, a proper evaluation of interspecific and interpopulation variations in these traits and their relation to resistance to insect herbivory and tolerance to damage has not been conducted yet in this system. In addition, there is interspecific variation in plant damage in natural sympatric populations. In mixed populations in humid areas, where *B. distachyon* diploids are more frequent, plant damage is higher in *B. hybridum* polyploids than in *B.distachyon*. If damage impacts fitness, then herbivory could contribute to maintaining the regional species differentiation observed in this complex (Manzaneda et al., 2012). Likewise, in mixed populations in arid areas, where *B. hybridum* polyploids are more abundant and competitively superior (Rey et al., 2017), herbivory could be a key ecological factor explaining coexistence of *B. distachyon* diploids and *B. hybridum* allotetraploids in dry contact zones whenever the impact of herbivory on fitness is lower for *B. distachyon*.

Here, we used the generalist insect herbivore *Locusta migratoria* (Orthoptera: Acrididae) to investigate covariation of leaf traits that are typically related to plant palatability/defense with insect plant damage and maternal fitness. We focused on palatability traits such as SLA, WC, and nitrogen content (all correlated positively to palatability, e.g., Schädler et al., 2003; Pontes et al., 2007), and traits such as silica content that increase resistance to insect damage (thus correlated negatively to palatability) (Frew et al., 2018; Biru et al., 2021). In particular, we addressed the following questions: (1) Do the three species of the complex vary in the amount of damage received and tolerance to damage? (2) Within each species, is there any variation in the extent of damage and tolerance to damage among populations or genotypes? (3) Do the three species vary in traits that are important for resistance to herbivory? If so, (4) which traits contribute the most to resistance and tolerance to insect herbivory?

By addressing these questions, we first investigated which plant traits drive differences in the insect herbivore response in the three *Brachypodium* ploidy lineages. Second, we answered whether insect herbivory may be an important ecological factor for *Brachypodium* species differentiation/coexistence across its range of distribution in the Iberian Peninsula (Manzaneda et al., 2012; Rey et al., 2017).

## MATERIALS AND METHODS

### Study system

The *Brachypodium distachyon* (Poaceae) species complex comprises three annual species—*B. distachyon*, *B. stacei*, and their allotetraploid derived *B. hybridum*—all native to the Mediterranean Basin and the Middle and Near East (Catalán et al., 2012). They inhabit a wide variety of climatic and ecological conditions from sea level to 2000 m a.s.l., frequently found in forest edges, natural xerophytic meadows, abandoned fields, and along roadsides (Manzaneda et al., 2012). Most populations include only one species, although sympatric populations of *B. hybridum* and *B. distachyon* exist (Manzaneda et al., 2012; López‐Alvarez et al., 2015). In those mixed populations, the relative frequency of diploids/polyploids is variable and dependent upon aridity; in humid areas, *B. distachyon* is more frequent, while *B. hybridum* is the most common in arid habitats (Manzaneda et al., 2012; Rey et al., 2017).

Flowering occurs between April and June, and all species are self‐pollinating (Vogel et al., 2009). Seeds mature and shed in the summer, then seeds germinate and seedlings emerge in October–November with the first autumn rainfalls. Grasshoppers (Orthoptera: Acrididae) are the main herbivores of *Brachypodium* plants in our study populations and typically feed on new seedlings during the early vegetative phase, significantly damaging plants in some populations (A. J. Manzaneda and P. J. Rey, unpublished data). Other herbivores such as Lepidoptera larvae cause negligible damage in our study populations (A. J. Manzaneda and P. J. Rey, personal observations).

### Plant material and growth conditions

We selected 119 genotypes from the *Brachypodium distachyon* species complex, including diploid and polyploid individuals, representing different populations (Appendix S1: Table S1). These genotypes were chosen to encompass genetic variation across populations: *B. distachyon*, 53 genotypes from 10 populations; *B. hybrdium*, 56 genotypes from 10 populations; *B. stacei*, 10 genotypes from three populations. The specific genotypes and their corresponding populations are detailed in Appendix S1 (Table S1). Selected genotypes originate from populations that are genetically differentiated (Martínez et al., 2018) and grow in contrasting climatic conditions (Appendix S1: Table S1). Nine mature seeds per genotype were placed in Petri dishes at 4°C for 4 d for cold stratification. Once germinated, they were individually planted and grown on a mix of perlite, sand, and standard soil (0.5:0.5:1 v/v) in randomized blocks. Each block consisted of one tray containing 48 pots (7 × 7 × 8 cm). Plants were grown under controlled conditions in a growth chamber (22°C, 16 h light and 8 h dark). All plants were watered with the same amount and frequency of watering, to ensure that all genotypes received consistent water availability, regardless of their original environment.

### Experimental procedure

After 4 weeks of growth, 357 plants (119 genotypes with three biological replicates each) were individually enclosed with one fourth‐star locust, the generalist herbivore *Locusta migratoria* (Orthoptera: Acrididae). Locust species are distributed widely from tropical to subarctic regions of Africa, Europe, and Asia, typically feeding on various Poaceae species, including cereal crops and grasslands (Massey et al., 2006 and references therein), and have been used previously to investigate palatability in grasses (e.g., Massey et al., 2006). Fourth‐instar locusts (average mass 600 mg; Animalia Co., Madrid, Spain) were starved for 12 h before a plant–locust pair was enclosed in a cylindrical acetate tube (diameter, 6 cm; height, 21 cm; 3 M, Madrid, Spain) with both ends open (Appendix S1: Figure S1). One end was inserted into the soil, and the other was covered with an organza bag (for a similar procedure: Manzaneda et al., 2010). After 24 h, locusts were removed, and plants were checked for damage. We used a 24‐h feeding window, which is within the standard range for acridid bioassays (typically 12–48 h; e.g., Massey et al., 2006; Hall et al., 2020; Brosemann et al., 2023). Preliminary tests indicated that this period consistently produced measurable damage while avoiding starvation artifacts, and all locusts fed actively during this interval. For each plant, we recorded the proportion of leaves with any herbivore damage, and the same person visually estimated the percentage of tissue removed per leaf (from 1% to 100%) to calculate the percentage of plant damage (PD) as the total number of leaves damaged multiplied by the average percentage of leaf damage/total number of leaves. Plants were then grown to maturity (ca. 8 weeks), and their aerial parts were harvested to analyze growth variables and reproductive output. For each plant, we recorded the number of spikes and seeds produced per plant and the reproductive biomass (mass of all seeds produced per plant) as estimates of maternal fitness.

### Plant trait measurements

Traits were measured for all damaged and undamaged plants (three biological replicates per genotype). Immediately after harvest, leaves for the plant were weighed on a precision scale to determine fresh mass (FW), then dried for 48 h at 70°C and weighed again (dry mass, DW). Water content (WC) was then calculated as 100 × [(FW − DW)/FW]. Specific leaf area (SLA) of the largest leaf was obtained from the ratio of leaf area to DW (m^2^ kg^−1^); leaf area was measured using a leaf area meter (LI‐3000C Portable Area Meter; LI‐COR, Lincoln, NE, USA). For quantifying leaf nutrients, leaves were dried for 48 h at 70°C, then ground using a mixer miller MM 200 and MM 400 (Retsch. Haan, Germany). Approximately 2 mg of the ground leaves (weighed using a high‐precision microbalance, ±0.001 mg), was then sealed in preformed tin capsules and loaded into the autosampler for elemental analysis. Carbon, nitrogen, and hydrogen were quantified via flash combustion using a Thermo Finnigan FlashEA1112 CHNS‐O elemental analyzer (Thermo Fisher Scientific, Waltham, MA, USA) and the manufacturer's procedures. Calibration was based on the synthetic standard sulfanilamide. Leaf silica content was determined using 1% Na_2_CO_3_ extraction at 85°C (Meunier et al., 2017). Briefly, approximately 33 mg of dried plant material were mixed with 40 mL of 1% Na_2_CO_3_ solution in a polypropylene bottle and placed in a shaker bath at 85°C and 100 rpm for 1 h., then 1 mL was removed from each sample bottle and placed into pre‐labelled Pyrex glass tubes containing 9 mL of 0.021 N HCl to neutralize Na_2_CO_3_. Dissolved Si (DSI) was then quantified using the molybdenum blue colorimetric method and Spectroquant reactants (Merck, Fontenay sous Bois, France). Absorption was measured at 820 nm after 3, 4, and 5 h to obtain a mean DSI; and a standard curve (*R* ^2^ > 0.999) was generated using dilutions of a 1000 mg/L standard Si solution CertiPUR (Merck, Rahway, NJ, USA).

### Statistical analyses

R version 3.5.3 (R Core Team, 2019) was used for all analyses. To analyze interspecific differences in plant damage and leaf functional traits, we fitted general linear mixed models with residual maximum likelihood (REML) estimation using the R package lme4 (Bates et al., 2015). Species was considered a fixed factor, and we included genotype nested within species as a random factor.

Because locust feeding was time‐limited, plant damage depends not only on plant quality or defense, but also on plant size, which varied significantly between species (*F*_2, 106_ = 5.19, *P* = 0.007; 18.38 ± 7.8, 15.2 ± 8.06, 11.6 ± 4.29 leaves developed at the beginning of the experiment for *B. distachyon*, *B. hybridum*, and *B. stacei*, respectively), we explored insect damage by including number of leaves as a covariate to account for interspecific and individual differences in plant size. Plant leaf number × species interaction term (assessing whether the way in which leaf number affected plant damage varies across species) was never statistically significant and eventually was not included in the models.

To analyze variation in plant damage among populations within each species, we fitted general linear mixed models with REML with population as a fixed factor and genotype nested within population as a random factor. Significance of fixed and random factors was evaluated using a likelihood ratio test (LRT) in the R package lmerTest (Kuznetsova et al., 2017). Effects of main factors and contrasts thereof were summarized by obtaining the least‐squares means (i.e., model estimated marginal means) using the function emmeans in the R package emmeans (Lenth, 2019).

To analyze variation in tolerance to damage between species, we used a linear mixed model, in which fitness response estimators were the total number of spikes, the total of number of seeds and the total seed biomass produced per each plant. Predictors in these models were species, plant damage, and their interaction and population nested within species and genotype nested within population as random factors. Tolerance of herbivore damage was then calculated as the slope of a regression of plant fitness on a continuous damage level (Wise and Carr, 2008). Likewise, we analyzed variation in tolerance to damage among *Brachypodium* populations (only for *B. distachyon* and *B. hybridum* populations; the low number of *B. stacei* was insufficient for this analysis) by fitting separated linear mixed models for each *Brachypodium* species. In these models, maternal fitness estimates were the dependent variables and plant damage, population and their interaction the predictors. Similarly, plant leaf number and genotype within population were also included as covariate and random factor, respectively. In this case, to encompass the entire damage spectrum (0%–100%) for each population, we included additional fitness data from undamaged plants for each genotype. In both cases, to obtain and compare estimates of slopes between species or populations, we used the function emtrends in R package emmeans (Lenth, 2019). When comparing tolerances among groups of plants, damage and performance need to be on the same multiplicative scale (Wise and Carr, 2008), we thus log‐transformed all fitness measurements.

To analyze interspecific and interpopulational trait variation, we used multivariate analyses in addition to the univariate analyses described above. A multivariate analysis of variance (MANOVA) was used to test the effects of species on overall trait variation. Next, we used a linear discriminant analysis using the R package DiscriMiner (Sanchez, 2013) to determine which traits best discriminated between *Brachypodium* species or among *B. hybridum* and *B. distachyon* populations.

Finally, to investigate predictors of plant damage and tolerance (i.e., estimates of slopes between species or populations), we carried out backward multiple regression analyses using the R package MASS and the function stepAIC, which selects the best model using the Akaike information criterion (AIC). In these analyses, we used genotypic means for C:N, WC, SLA, silica content and their interactions, which were regressed against genotypic plant damage and tolerance separately. Because *B. distachyon* leaf traits were significantly differentiated from those of *B. hybridium* and *B. stacei* (see Results), we fitted separate regression models for *B. distachyon* genotypes.

Response variables were transformed when necessary (log or angular transformation in the case of proportion‐based data).

## RESULTS

### Variation in plant damage

**Figure 1:**
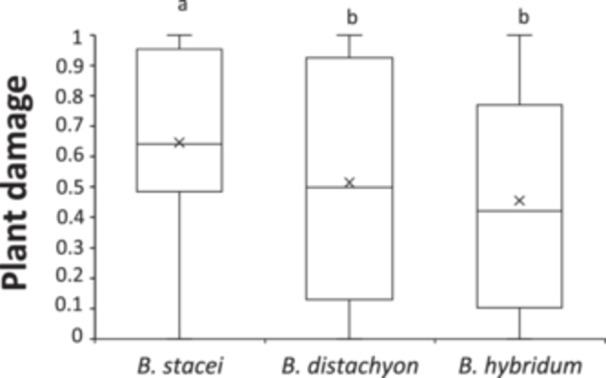
Variation in plant damage (proportion of the available leaves consumed during bioassays) among the three species of the *Brachypodium distachyon* species complex. Box plots depict the whole variation of plant damage for each species. Horizontal lines are the median; X is the estimated marginal mean. Different letters indicate a significantdifference (*P* < 0.05) in post hoc contrasts.

Plant damage varied significantly between *Brachypodium* species (*F*_2, 112_ = 3.27, *P* = 0.042, Figure 1). Thus, *B. stacei* plants received 1.44 and 1.27 times more damage than *B. hybridum* and *B. distachyon* plants (Figure 1) respectively. Plant damage varied significantly among genotypes within species (LRT = 14.96, df = 1, *P* < 0.0001) and was significantly influenced by the number of leaves (estimate ± SE: –0.016 ± 0.002; *F*_1, 262_ = 45.4, *P* < 0.0001). For *B. distachyon* and *B. stacei*, overall plant damage levels did not vary significantly among populations (*B. distachyon*: *F*_11, 37_ = 1.11, *P* = 0.382; *B. stacei*: *F*_2, 7_ = 0.49, *P* = 0.63; Appendix S1: Figure S2). For *B. hybridum*, variation in plant damage among populations was marginally significant (*F*_9, 148_ = 2.54, *P* = 0.09; Appendix S1: Figure S2).

### Variation in tolerance to herbivory

**Figure 2:**
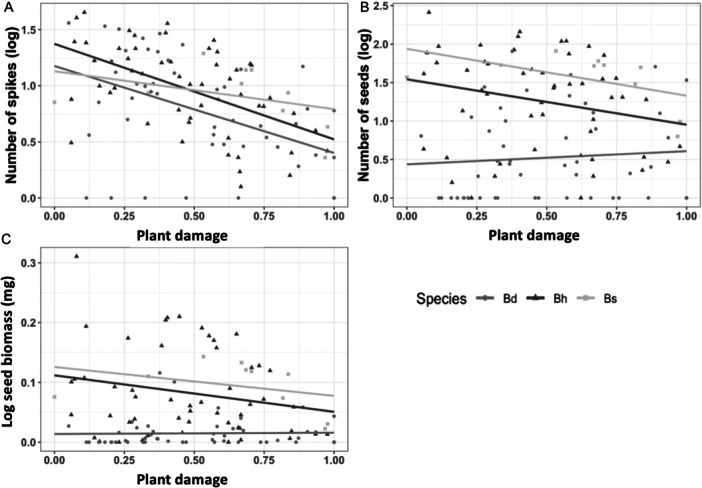
Variation in fitness across insect herbivory damage in three species of the *Brachypodium distachyon* species complex. (A) Number of spikes, (B) number of seeds, (C) seed biomass. Lines depict the linear fit between the two variables for each species.

Herbivory damage affected significantly fitness, although its effect depended on fitness estimator and species (Table 1). Thus, the number of spikes was reduced significantly in relation to plant damage (estimate ± 1 SE: –0.52 ± 0.11; Figure 2) and was consistent between species, as indicated by the lack of significance of the interaction plant damage × species (Table 1). For number of seeds and total biomass, influence of plant damage was dependent on the species (Table 1, Figure 2), suggesting interspecific variation in tolerance to damage for these later fitness components. Thus, *B. distachyon* was significantly more tolerant to damage than *B. hybridum* and *B. stacei* (Appendix S1: Table S2, Figure 2), showing that despite *B. distachyon* plants are less vigorous (fewer spikes and seeds and less seed biomass) than their relatives, they were able to tolerate in a large extent herbivory damage. Finally, population and genotype also influenced fitness (Table 1), reflecting interpopulational and intergenotypic differences in vigor.

**Table 1:** Summary results of the general linear mixed model testing the effects of plant damage, species and their interaction on three different maternal fitness components in the *Brachypodium distachyon* species complex. A significant interaction of plant damage × species denotes interspecific variation in tolerance to damage.

	No. of spikes			Total no. seeds			**Seed biomass**		
**Effects**	**df**	***F***	***P***	**df**	***F***	***P***	**df**	***F***	***P***
Plant damage	1, 223	31.2	**<0.0001**	1, 281	18.7	**<0.0001**	1, 268	22.45	**<0.0001**
Species	2, 53	1.67	0.198	2, 63	22.1	**<0.0001**	2, 70	23.5	**<0.0001**
Plant damage × Species	2, 249	0.59	0.55	2, 280	7.96	**0.0004**	2, 276	9.32	**0.0001**

Random effects	df	LRT	*P*	df	LRT	*P*	df	LRT	*P*
Population (Species)	1	10.01	**0.0015**	1	4.16	**0.041**	1	3.04	0.08
Genotype (Population)	1	14.34	**0.0001**	1	52.94	**<0.0001**	1	40.29	**<0.0001**

*Note*: Significant *P* values (<0.05) are in bold.

**Figure 3:**
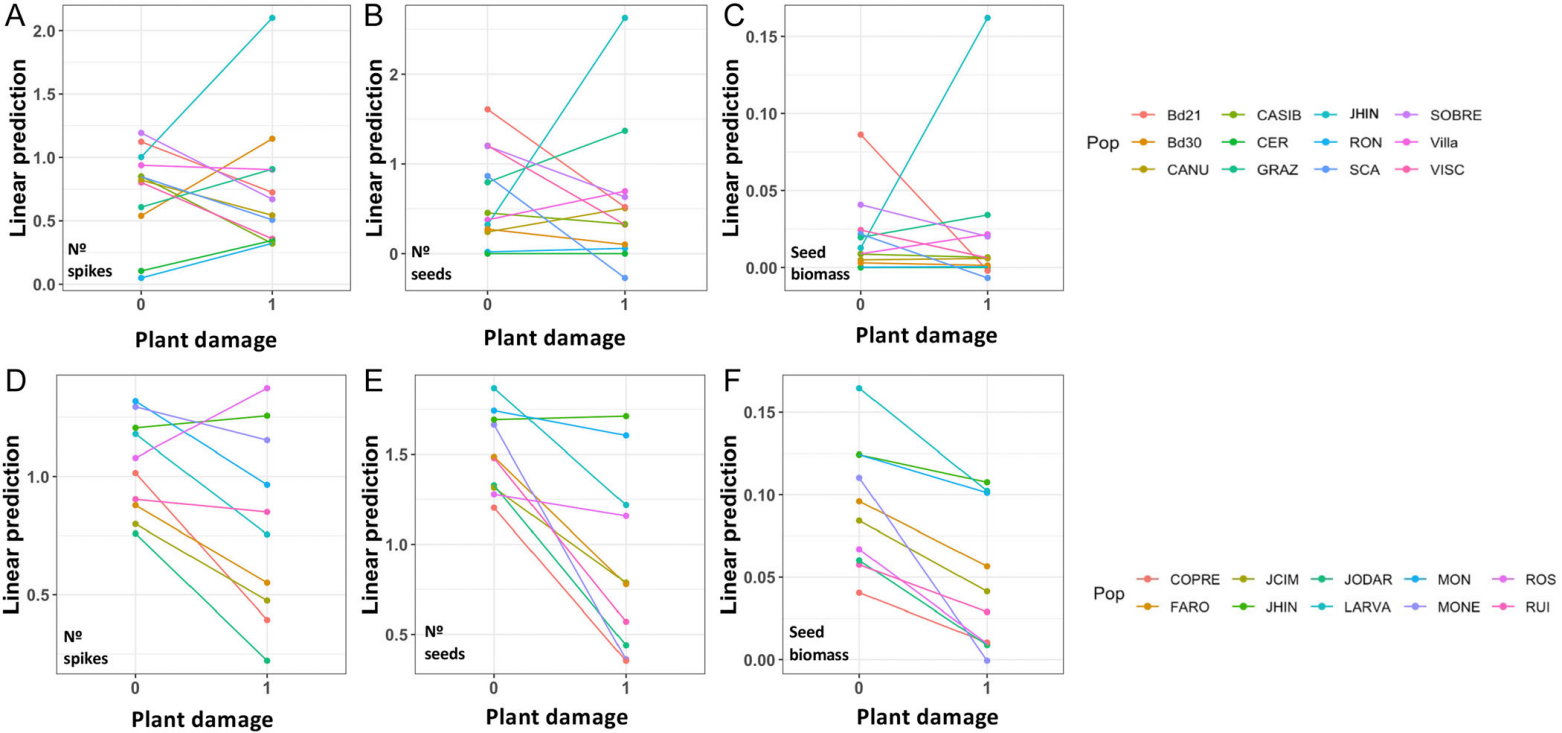
Tolerance slopes for populations of (A–C) *Brachypodium distachyon* and (D, E) *B. hybridum*. Graphs show the linear prediction of fitness variation across the range of plant damage (0–1) for three maternal fitness components (A, D, number of spikes; B, E, number of seeds; C, F, total seed biomass).

At the population level, variation in tolerance to damage among populations was detected only for *B. distachyon* populations, not for *B. hybridum* (Appendix S1: Tables S3, S4; Figure 3). Thus, in the case of *B. distachyon* for all fitness components examined, we detected populations that remained unaffected by damage, that were able to overcompensate herbivory damage, or that fitness decreased accordingly to increases in damage (Figure 3A–C). Contrarily, for *B. hybridum* populations, overall, plant damage reduced plant fitness to a greater or lesser extent (Figure 3D–F).

### Variation in functional traits

For *B. distachyon* and *B. hybridum*, silica content and C:N were negatively correlated, and SLA and WC positively associated (Appendix S1: Table S5). In *B. stacei*, traits appeared uncorrelated (Appendix S1: Table S6).

**Figure 4:**
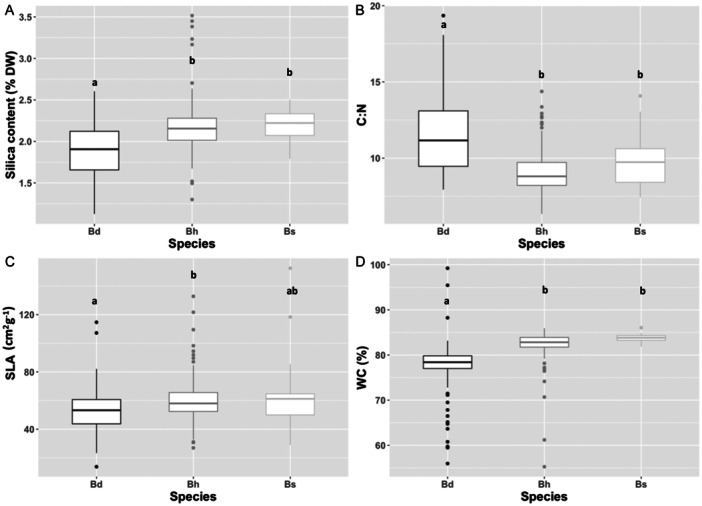
Variation in (A) silica content, (B) C:N ratio, (C) specific leaf area (SLA), and (D) water content (WC) between the three species of the *Brachypodium distachyon* species complex. Different letters indicate significant interspecific pairwise differences (*P* < 0.05) in post hoc contrasts tests. Bd, *B. distachyon*; Bh, *B. hybridum*; Bs, *B. stacei*.

Overall, leaf functional traits varied between species (MANOVA result: Wilk's *λ* = 0.57, *F*_8,606_ = 24.34, *P* < 0.0001). Thus, linear discriminant analysis showed that *B. distachyon* plants were significantly differentiated from *B. hybridum* and *B. stacei* in leaf functional traits related to herbivore response (Wilk's *λ* = 0.64, *P* < 0.0001; Appendix S1: Figure S3). Silica content was the trait with the highest weight for the discriminant variables DF1 and DF2 (Table S7), accounting for a total of 81.25% and 18.75% of between‐species variation, respectively. Univariate analysis performed on each trait confirmed such interspecific variation (*F*_2, 103_ = 13.41, *P* < 0.0001; *F*_2, 104_ = 28.16, *P* < 0.0001; *F*_2, 104_ = 3.49, *P* = 0.034; *F*_2, 104_ = 25.24, *P* < 0.0001; results for silica content, C:N, SLA, and WC respectively; Figure 4). In particular, *B. distachyon* showed a lower silica content, water content and SLA, and a higher C:N ratio than *B. hybridum* and *B. stacei* (Figure 4).

At the population level, linear discriminant analysis indicated the existence of interpopulation differentiation in leaf functional traits both in *B. hybridum* (Wilk's *λ* = 0.4, *P* < 0.0001) and *B. distachyon* (Wilk's *λ* = 0.28, *P* < 0.0001) (Appendix S1: Figure S4). Silica content was again the trait with the highest weight for the main discriminant variable DF1 accounting for a total of 49% and 47.1% of interpopulational variation, for *B. hybridum* and *B. distachyon*, respectively (Appendix S1: Table S8). For *B. distachyon*, the C:N ratio was also significant for discriminating among populations accounting for a 31% of interpopulational variation (Appendix S1: Table S8). Thus, JHIN, LARVA, and MON were significantly differentiated from the rest, essentially in terms of leaf silica content (Appendix S1: Figure S4), whereas SCA, VILLA, and VISC appeared differentiated from the rest of *B. distachyon* populations according to silica content and C:N (Appendix S1: Figure S5).

Univariate analyses showed that, for both species, all traits but SLA varied significantly among populations (*B. hybridum*: *F*_9, 46_ = 13.4, *P* < 0.0001; *F*_9, 46_ = 28.16, *P* = 0.001; *F*_9, 46_ = 1.39, *P* = 0.219; *F*_9, 46_ = 2.09, *P* = 0.049; *B. distachyon*: *F*_9, 31_ = 9.56, *P* < 0.0001; *F*_9,3 1_ = 6.5, *P* < 0.0001; *F*_9, 31_ = 1.35, *P* = 0.252; *F*_9, 31_ = 2.36, *P* = 0.036; results for silica content, C:N, SLA and WC respectively; Appendix S1: Figure S5). For both species, interpopulational trait variation was not related to any geographical and/or environmental parameter (*P* > 0.05 data not shown).

### Plant resistance and tolerance predictors

**Figure 5:**
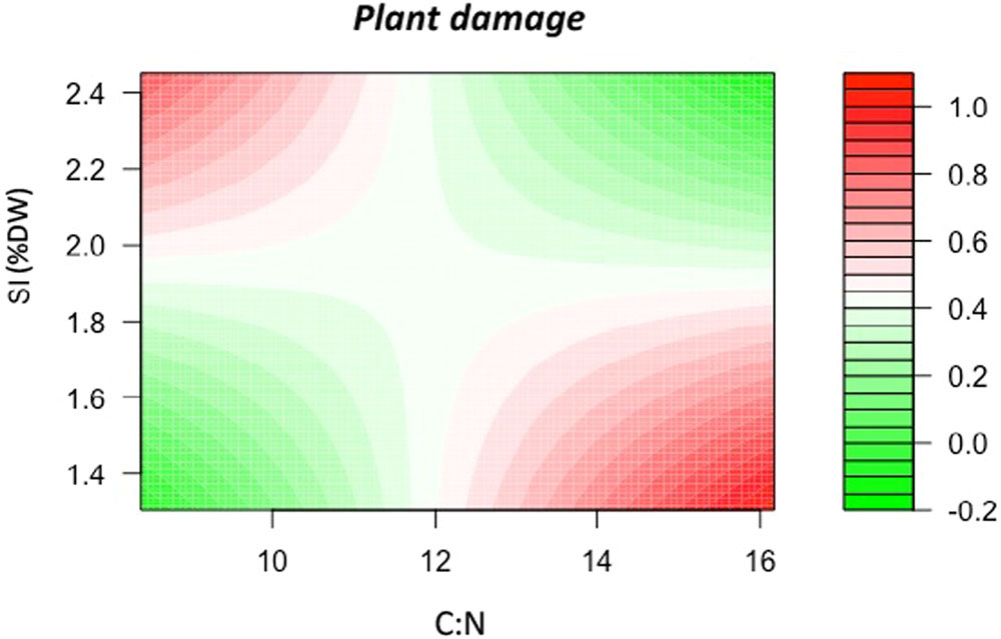
(A) Contour plot depicting the interactive effect of leaf silica content (% of dry mass) and C:N ratio on plant damage in *B. distachyon* genotypes.

For *B. distachyon* genotypes, we found a significant association between C:N, silica content and plant damage, which was interdependent and complex (Table 2, Figure 5). Thus, plant damage was function of a combination of silica content and C:N yielding a saddle point‐type surface (Figure 5), suggesting the existence of nonlinear relationships underlying the association between C:N, silica content and plant damage. In fact, the association between plant damage and the quadratic terms of C:N and silica content was statistically significant for the case of C:N (0.238 ± 0.009, *F*_1, 36_ = 5.95, *P* = 0.019; Appendix S1: Figure S5), and marginally significant for silica content (0.824 ± 0.479, *F*_1, 36_ = 2.96, *P* = 0.09; Appendix S1: Figure S5). For *B. hybrdium* and *B. stacei* genotypes, only SLA was marginally associated with plant damage (Table 2).

**Table 2:** Summary results of the backward multiple regression analysis performed between leaf traits and plant damage for *Brachypodium* plants.

**Effect**	**Estimate (SE)**	**df**	***F***	***P***
***B. distachyon***				
Silica content	2.93 (0.85)	1, 35	0.24	0.626
C:N	0.48 (0.13)	1, 35	0.52	0.474
Silica content × C:N	**–0.25 (0.07)**	1, 35	4.25	**0.0014**
***B. hybridum/B. stacei***				
Specific leaf area	0.004 (0.002)	1, 61	3.39	0.07

*Note*: Significant coefficients (*P* < 0.05) are in bold.

**Figure 6:**
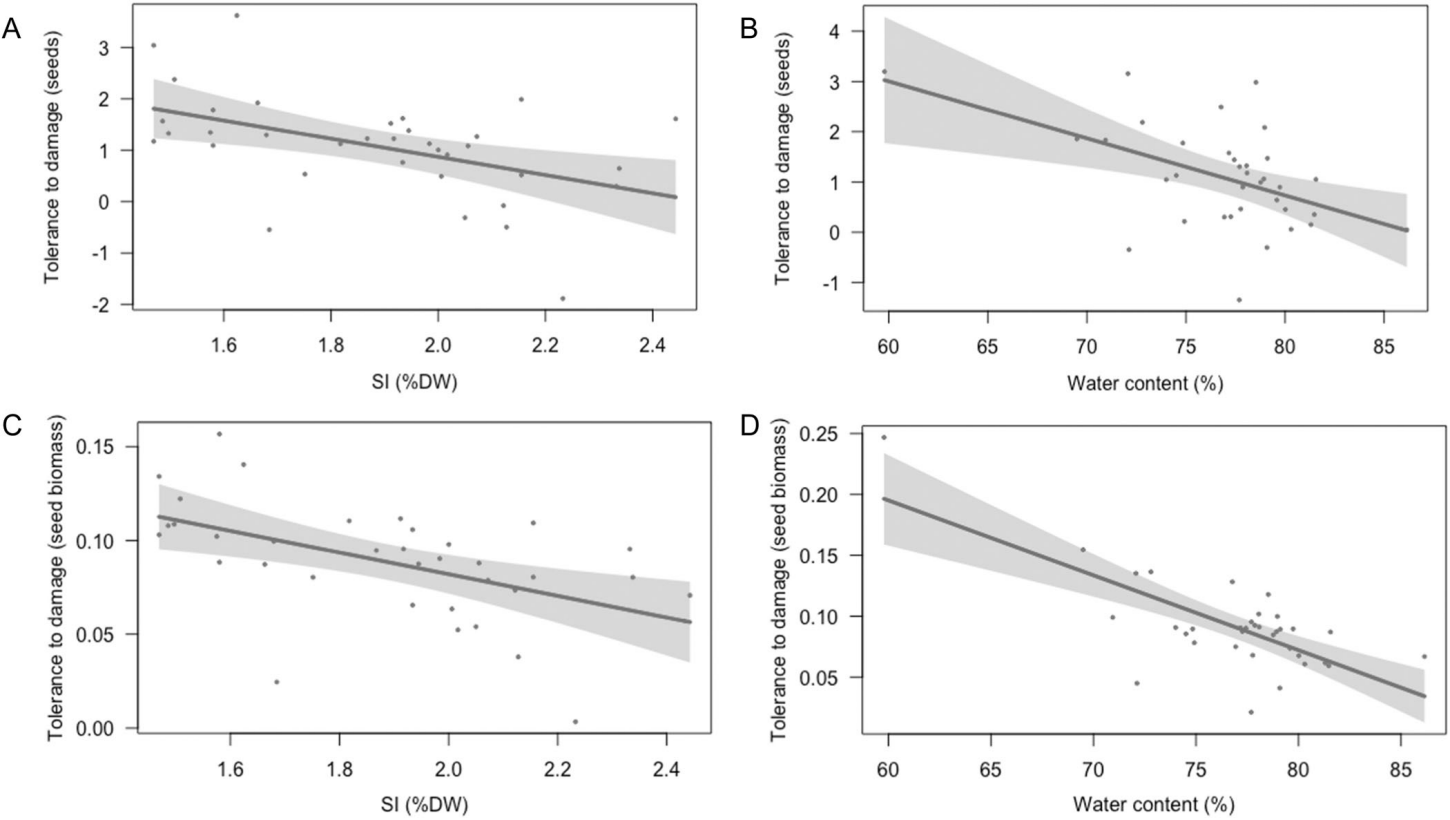
Relationships between silica (SI, % of dry mass [DW]) and water content and tolerance to damage across *Brachypodium distachyon* genotypes. Tolerance estimates in A and B are from slopes between plant damage and the number of seeds (multiple *R* ^2^ = 0.39, *F*_4, 30_ = 4.9, *P* = 0.0036). Tolerance estimates in C and D are from slopes between plant damage and seed biomass (multiple *R* ^2^ = 0.61, *F*_4, 30_ = 12.03, *P* < 0.0001). Dashed lines are 95% confidence intervals.

Regarding tolerance, for *B. distachyon*, silica and water content were negatively linked to tolerance as estimated from seed number and seed biomass tolerance estimators (Table 3, Figure 6), indicating that plants with higher silica and water content also were less tolerant of damage. For *B. hybridum* and *B. stacei*, no leaf trait was significantly related to tolerance (*P* > 0.05 in all cases).

**Table 3:** Summary results of the backward multiple regression analysis performed between leaf traits and plant tolerance for *Brachypodium distachyon* plants.

	No. of spikes				No. of seeds				Seed biomass			
Effect	Estimate (SE)	df	*F*	*P*	Estimate (SE)	df	*F*	*P*	Estimate (SE)	df	*F*	*P*
Silica content	0.7 (0.62)	1, 30	0.13	0.718	**–1.77 (0.57)**	**1, 30**	**9.68**	**0.0039**	**–0.067 (0.02)**	**1, 30**	**11.5**	**0.0018**
C:N	0.19 (0.09)	1, 30	3.51	0.07	–0.04 (0.11)	1, 30	2.28	0.141	–0.003 (0.003)	1, 30	0.71	0.403
Water content	**–**0.04 (0.03)	1, 30	0.34	0.564	**–0.11 (0.03)**	**1, 30**	**10.85**	**0.0024**	**–0.006 (0.001)**	**1, 30**	**35.29**	**<0.0001**
SLA	0.02 (0.01)	1, 30	1.63	0.21	–0.006 (0.017)	1, 30	0.25	0.61	0.0003 (0.003)	1, 30	1.004	0.324

*Note*: Significant coefficients (*P* <0.05) are in bold.

Abbreviation: SLA, specific leaf area.

## DISCUSSION

Results from our bioassay indicate that insect herbivory had a significant impact on maternal fitness in the *B. distachyon* species complex, denoting the potential of insect herbivores as selection agents for *Brachypodium* genotypes. The impact of herbivory on fitness was, however, largely dependent on species and the fitness estimator considered. Findings from this study did not support the hypothesis that polyploid lineages have an enhanced herbivory response in this species complex since *B. distachyon* diploid plants were more tolerant to damage and similarly resistant to herbivory than their polyploid relatives. Overall, differences in resistance appeared associated with differentiation in functional traits, especially in C:N ratio and silica content. Finally, for *B. distachyon*, differences in tolerance to damage were linked negatively to silica and water content variation, which suggests the existence of trade‐offs in tolerance andresistance traits in this species.

### Herbivory response in *B. distachyon* species complex

Evolutionary theory predicts that polyploidy may cause alterations in physiology, morphology, and gene expression that may impact resistance to herbivores (e.g., Lou and Baldwin, 2003; Hull‐Sanders et al., 2009; Segraves and Anneberg 2016, but see Gaynor et al., 2020). Therefore, if polyploidy confers resistance, less damage on polyploid populations would be expected. Results from our assay do not support this expectation since plant damage of the allotetraploid *B. hybridum* was equivalent to one of its diploid parentals, *B. distachyon*. Intriguingly, such equivalence in the amount of plant damage received between *B. hybridum* and *B. distachyon* did not arise from a similarity between these two species in functional traits related to herbivore resistance; instead, there was a higher correspondence in such functional traits between *B. hybridum* and the other diploid parental species that showed higher damage levels, *B. stacei* (Appendix S1: Figure S3). Although herbivore preference patterns among ploidies are not clearly established, differential insect selection on different ploidies has been documented in some systems. For example, in *Cardamine pratensis*, gall midge attacks are restricted to the higher ploidies (Arvanitis et al., 2010). Contrarily, in the genus *Leucanthemum*, resistance to the specialist root herbivore *Dichrorampha aeratana* consistently increases with increasing plant ploidy level (Stutz et al., 2016). In the present study, plant damage received was similar between *B. hybridum* allopolyploids and *B. distachyon* diploids, while plant damage recorded for *B. stacei* diploids was about 30% higher compared to its relatives.

These results contrast with previous field observations in humid Iberian mixed populations, where *B. hybridum* allotetraploids are more frequently attacked by insect herbivores than *B. distachyon* diploids (the proportion of plants with any sign of leaf damage is 47.5% vs. 26.5%, *N* = 819, data observed for *B. hybridum* and *B. distachyon*, respectively; A. J. Manzaneda and P. J. Rey, unpublished data). Such discordance may reflect effects derived from the particular ecological context in these populations influencing herbivore response, such as the insect herbivore species, the relative spatial arrangement of plant species, and/or the level of plant competition. However, a deeper evaluation of the insect herbivory pattern across multiple natural contact diploids/polyploids populations is still lacking and further research is needed to verify herbivore preference in this system.

In any case, for all species, intraspecific variation in plant damage appeared more important than interspecific variation. In particular, genetic variation at the intra‐ and interpopulational scale accounted for almost one third of the variation of damage on plants. Interindividual variation in plant damage at different spatial scales has been described repeatedly in many systems (e.g., Maddox and Root, 1987; Adler et al., 1995; Ivey et al., 2009; Schranz et al., 2009; Martínez et al., 2018) and seems to be the case here also. Causes underlying intergenotypic variations in plant damage within populations are beyond the scope of this study but is likely related to interindividual variation in traits that confer resistance to herbivores in this system (C:N ratio and silica content). In any case, because herbivory affected fitness, existence of significant intergenotypic variation in plant damage could result in herbivore‐mediated trait selection if herbivore pressure is sustained (Agrawal et al., 2012), which occurs in several Iberian *Brachypodium* populations (A. J. Manzaneda, personal observations).

Beyond resistance, plants may evolve alternative defensive strategies that do not influence the amount of damage received but maintain fitness, such as tolerance (Strauss et al., 2002; Fornoni, 2011). In the context of polyploidy, few studies have compared the reaction norms of maternal fitness across different levels of damage between the different cytotypes within heteroploid species (but see Boalt et al., 2010; König et al., 2014). Our results do not back the expectation of enhanced tolerance for polyploid lineages since *B. distachyon* diploid plants showed higher tolerance to insect damage than *B. hybridum* allotetraploids in two of the three fitness components analyzed (Figure 2). Similarly, when compared to *B. stacei*, the other diploid parent, tolerance to damage in *B. hybridum* plants was lower or similar (Figure 2). Our results indicate that tolerance variation depended upon the fitness component analyzed, the species and the specific genotype. Thus, for the early fitness component (the number of spikes yielded per plant), herbivory damage affected fitness of all species in a similar manner, i.e., a reduction of the reproductive potential around 50%. For this fitness component, intergenotypic variation was more important than interspecific one, suggesting extensive genetic variation in tolerance to damage. For later fitness components (the number of seeds and seed biomass), *B. distachyon* was the only species that could compensate herbivory damage (Figure 2), whereas for *B. stacei* and *B. hybridum*, herbivory reduced the reproductive potential of plants about 74% and 78%, respectively. Genetic heterogeneity in response to damage is common at different levels (Strauss and Agrawal, 1999; Stevens et al., 2007; Manzaneda et al., 2010), ranging from mortality to overcompensation. Our results support this view, with *B. distachyon* genotypes, being, overall, able to overcome plant damage with a variable magnitude (with the exception of Bd21 and SCA lines, Figure 3), whereas fitness of *B. hybridum* and *B. stacei* genotypes is consistently decreased by insect damage (Figure 3). Traits that modify physiological processes such as photosynthetic activity and growth, phenology, and/or the use of stored nutrient typically underlie variation in tolerance to herbivory damage (Mitchell et al., 2016). Here, for *B. distachyon*, we detected a negative trend between tolerance and constitutive silica and water content, indicating that plants with higher silica content and water content tend to have less tolerance to damage (see the following section for further discussion on this relationship). For *B. hybridum* and *B. stacei*, we did not detect any relationship with leaf traits that could explain variation in tolerance.

Although our study did not find any evidence that polyploidy may enhance herbivore response in this species complex, the findings from this study may be relevant for explaining the coexistence of *B. distachyon* diploids and *B. hybridum* allotetraploids in contact zones and the establishment of allotetraploids populations. Previously, we showed a higher competitive advantage of *B. hybridum* allotetraploids compared with their parental *B. distachyon* diploids in natural mixed populations in southern Spain, forecasting in addition the exclusion of *B. distachyon* diploids, especially from contact zones situated at dry localities (Rey et al., 2017). However, insect herbivory could reduce the competitive ability of allotetraploids directly by diminishing the reproductive potential of *B. hybridum* allotetraploids and indirectly allowing *B. distachyon* diploids to counterbalance fitness differences between species derived from a higher vigor of polyploids. Consistent with this idea is the fact that (1) despite predictions of cytotype competitive exclusion (Rey et al., 2017), mixed populations of *B. hybridum* allotetraploids and *B. distachyon* diploids are frequent in southern of the Iberian Peninsula (21% of sampled Iberian populations; Manzaneda et al., 2012); and (2) in natural populations, *B. hybridum* allotetraploids are more frequently attacked by herbivores than *B. distachyon* diploids. Many studies have already demonstrated the interactive effect of herbivory and plant competition in many systems (e.g., Hambäck and Beckerman, 2003; Hanley and Sykes, 2009; Agrawal et al., 2012). In any case, to determine whether insect herbivory is a stabilizing factor that promotes species coexistence in this system requires experimental verification in natural populations.

### Functional traits underlying herbivory response in *B. distachyon* species complex

Differentiation in ecologically important characters between ploidies in heteroploidy species is common for many functional and physiological traits (see Manzaneda et al., 2015; Soltis et al., 2016; Martínez et al., 2018). For plant defense‐related traits, however, such differentiation is not always straightforward, and there is no consistent trend between ploidy and defensive traits (Gaynor et al., 2020). Here, our results do not support the hypothesis that ploidy mediates differentiation of herbivore‐response traits in this species complex. Thus, we have confirmed that traits related to leaf palatability and resistance of *B. hybridum* allotetraploids and *B. stacei* diploids are similar, while these traits were clearly differentiated for *B. distachyon* diploids from the other two species, especially in terms of leaf silica content. Silicon is well recognized for alleviating diverse forms of abiotic and biotic stress in plants including deterring herbivore (Frew et al., 2018), especially for grasses, in which silicon in the form of opaline phytoliths in the leaves, deters feeding, perhaps due to its abrasiveness, and reduces digestibility (Massey et al., 2006). Findings from our bioassay do not show, however, a direct link between silica content and plant damage. At the species level, plant damage is not associated with silica content; the species with the highest leaf silica content, *B. stacei*, also had the most damage. Likewise, the species with a lowest silica content, *B. distachyon*, had the same amount of damage as *B. hybridum*, which had ca. 25% more leaf silica. At the intraspecific level, only for *B. distachyon* did we detect a significant association between plant damage and leaf silica, although the relationship was dependent upon the C:N ratio (Figure 5) because the traits appeared to be negatively correlated (Appendix S1: Table S5). Thus, our results indicated that covariation in silica content and C:N ratio explained, in a nonlinear manner, variation in damage for this species. Thus, genotypes with more damage had a higher C:N ratio but lowest leaf silica, and those that had a low C:N ratio accumulated more silica. In other words, greater resistance to insect damage results from the combination of high leaf silica content and elevated C:N ratio (low palatability) and, surprisingly, also from a high palatability combination resulting from a low C:N ratio and low leaf silica (Figure 5). Though higher resistance to damage concordant with low palatability is expected, our results also suggest that locusts were able to balance between the leaf N proportion (more nutritious) and leaf silica content, probably due to the ability of orthoptera to cope with mechanical damage from silicified cells and trichomes (Hall et al., 2020). Similar to the findings of Hall et al. (2020), our results did not show any association between silica content and leaf mechanical traits, such as the specific leaf area and water content, which may explain the limited impact of silica content on deterring herbivory in this species complex. However, variability in the impact of silicon on the herbivory pattern that we found is in contrast to previous studies on *B. distachyon*, which have shown a direct negative association between silica leaf content or silica cell density variation and herbivore response (Hall et al., 2020; Biru et al., 2021). Discrepancies between the present results and previous findings may arise, perhaps, from the use of different lines in the experimentation, the specific identity of insect herbivores, and/or the silicon availability in the soil during plant growth (e.g., Hall et al., 2020).

Although we focused on ploidy differences, the variation in genome size within the complex suggests nucleotypic effects could also influence phenotypic traits. Diploid ancestors differ slightly in genome size (Catalán et al., 2012), and the genome of the allotetraploid *B. hybridum* exceeds 1.1 pg. However, our results show that neither ploidy level nor genome size predicts herbivory resistance or tolerance; *B. hybridum* is not more defended or tolerant than *B. distachyon* diploids, nor do *B. stacei* and *B. hybridum* differ in herbivory traits. Thus, a larger genome does not necessarily enhance herbivore response. However, genome size affects cell traits and resource allocation (Bennett, 1987; Knight et al., 2005), potentially influencing physiological traits. *B. distachyon*'s slightly larger genome correlates with differences in C:N ratio, water content, and silica accumulation, indicating genome size may contribute to trait divergence independently of ploidy. Considering genome size therefore offers a broader view of herbivory‐related trait variation across *Brachypodium*, even if it does not increase resistance or tolerance.

Lastly, for *B. distachyon* and *B. hybridum*, we also detected population trait differentiation. However, we did not find any link between trait variation, environment and/or geography across our study sites (results not shown), which contrasts with our previous findings for other functional traits which covaried with aridity (Manzaneda et al., 2015; Martínez et al., 2018).

The existence of positive genetic correlations between tolerance and resistance may promote the allocation of resources simultaneously to both tolerance and resistance, and in turn, the joint evolution of tolerance and resistance to damage (Núñez‐Farfán et al., 2007; Manzaneda et al., 2010). On the contrary, the negative genetic correlations between tolerance and resistance indicate the presence of allocation costs, which are thought to maintain the existing levels of genetic variation in tolerance within species (Núñez‐Farfán et al., 2007). Here, we detected a relationship between tolerance and resistance only for *B. distachyon*. Thus, for this species, we found significant negative genetic correlations between tolerance and variation in constitutive silica and water content, both leaf mechanical traits. Regarding silica content, the more relevant trait for resistance to damage in this species, we detected that genotypes with high leaf silica content are associated with a lower ability to compensate insect damage compared to genotypes that accumulated less silica. This result is relevant for the evolution of resistance in this species because the evolution of resistance to damage may be constrained for genotypes that have low palatability and rely partially on the accumulation of high levels of silica. Simultaneously, natural selection could favor the evolution of those genotypes that show intermediate or lower amount of silica content. There are two plausible and non‐exclusive explanations that may underlie the existence of such a trade‐off: (1) Silica accumulation is more costly than typically assumed and thus could affect regeneration of tissues and reproduction after damage. (2) Because silica content and C:N ratio are negatively linked, genotypes with high levels of silica also have relatively low levels of constitutive carbon, which may compromise regrowth after damage. In relation to water content, our results suggest that genotypes with high water content (higher palatability) are also less tolerant to damage than those with lower water content and thus less palatable, which could result in an adaptive advantage for less‐palatable genotypes, especially if such low palatability is not dependent on silica content. Importantly, most of the functional traits analyzed here have a plastic nature, so their expression may be affected by the ecological context such as drought and/or competition (Agrawal et al., 2008; Martínez et al., 2018), that may in turn affect the outcome of the plant response to herbivory (Grinnan et al., 2013).

## CONCLUSIONS

Here we analyzed for the first time the variation in the herbivory response in the *Brachypodium distachyon* species complex in the context of ploidy and trait variation from multiple populations. Our study does not support the notion that polyploidy may enhance herbivore response in this species complex because the polyploids did not have higher resistance and/or superior tolerance to damage compared to the diploids. A limitation of this study, however, is that our data do not allow us to infer precisely whether the trait expression and patterns of herbivore response described here result from whole‐genome duplication per se, ancient interspecific hybridization, or subsequent evolution after polyploid formation. Although the fact that trait expression and tolerance to herbivory damage of *B. hybridum* polyploids resembled *B. stacei* diploid parents is concordant with expectations from hybridization, our results point out the potential of Acrididae species—principal natural enemies of *Brachypodium* in our populations—as agents of selection of *Brachypodium* genotypes and for herbivore‐response traits, which may have promoted trait and herbivory response differentiation across *Brachypodium* populations. Thus, given that interpopulational and intergenotypic variation in the herbivore response was relevant, resistance and tolerance variation should rather be related to the herbivory history of the populations rather than to effects related to polyploid formation (Boalt et al., 2010). Future studies should examine the extent of variation in herbivory in natural populations, with special attention to contact zones in sympatric populations, and evaluate the selection pattern of herbivores within and between species. In any case, because insect herbivory had a greater effect on *B. hybridum* allotetraploids, which are superior to *B. distachyon* diploids in competitive ability (Rey et al., 2017), insect herbivory may act as a stabilizing mechanism that promotes species coexistence in contact zones. In addition, comparative studies combining synthetic, newly synthesized allo‐ and autotetraploids with an examination of natural variation across genotypes with different ploidy level, are now possible in this system because stable synthetic allotetraploids are already available in the *Brachypodium* species complex (Dinh Thi et al., 2016) and will help to answer the elusive question of what genetic and evolutionary causes determine in a larger extent phenotypic expression of ecologically important traits in allopolyploid lineages (Soltis et al., 2016).

## AUTHOR CONTRIBUTIONS

A.J.M.A., P.J.R., A.F.O., and L.M.M. designed and planned this research. L.M.M. and T.S. conducted the bioassay. A.J.M.A. and L.M.M. analyzed the data and wrote the paper with the feedback of P.J.R. All authors read the paper and made critical comments on early versions of the manuscript.

## Supporting information


**Appendix S1.** Supplemental tables and figures.
**Figure S1:** Details of the method followed to infest *Brachypodium* plants with locusts.
**Figure S2:** Variation in plant damage (proportion of the available leaves consumed during bioassays) among populations of the *Brachypodium distachyon* species complex.
**Figure S3:** Position of the three species of the *Brachypodium distachyon* species complex over the plane defined by the first two discriminant factors DF1 and DF2, obtained from discriminant analyses of four functional traits.
**Figure S4:** Position of the *B. hybridum* populations and *B. distachyon* over the plane defined by the first two discriminant variables DF1 and DF2, obtained from discriminant analyses of four functional traits.
**Figure S5:** Variation in functional traits silica content, C:N ratio, specific leaf area, and water content among *B. hybridum* and *B. distachyon* Iberian populations.
**Figure S6:** Relationships between the C:N ratio and plant damage across *B. distachyon* genotypes. and relationships between silica content and plant damage across *B. distachyon* genotypes.
**Table S1:** Brachypodium ID accessions and geographical origin of the plants included in the study.
**Table S2:** Regression coefficients of the relationship between plant damage and fitness for each *Brachypodium* species.
**Table S3:** Summary results of the general linear mixed model testing the effects of plant damage, population, and their interaction on three different maternal fitness components in *Brachypodium distachyon*.
**Table S4:** Summary results of the general linear mixed model testing the effects of plant damage, population, and their interaction on three different maternal fitness components in *Brachypodium hybridum*.
**Table S5:** Phenotypic correlations (Pearson's product–moment correlations) between leaf functional traits for *Brachypodium distachyon* and *Brachypodium hybridum*.
**Table S6:** Phenotypic correlations (Pearson's product–moment correlations) between leaf functional traits for *Brachypodium stacei*.
**Table S7:** Coefficients of the linear discriminant functions (DFs) of the four functional traits included in the discriminant analysis conducted at species level.
**Table S8:** Coefficients of the linear discriminant functions (DFs) of the four functional traits included in the discriminant analysis conducted at population level.

## Data Availability

All data supporting the analyses presented in this paper are archived and publicly available at the following link: https://doi.org/10.5281/zenodo.18060483.

## References

[ajb270176-bib-0001] Adler, L. S. , J. Schmitt , and M. D. Bowers . 1995. Genetic variation in defensive chemistry in *Plantago lanceolata* (Plantaginaceae) and its effect on the specialist herbivore *Junonia coenia* (Nymphalidae). Oecologia 101: 75–85. 28306979 10.1007/BF00328903

[ajb270176-bib-0002] Agrawal, A. A. 2011. Current trends in the evolutionary ecology of plant defence. Functional Ecology 25: 420–432.

[ajb270176-bib-1001] Agrawal, A. A. , A. C. Erwin , and S. C. Cook . 2008. Natural selection on and predicted responses of ecophysiological traits of swamp milkweed (Asclepias incarnata). Journal of Ecology 96: 536–542.

[ajb270176-bib-0003] Agrawal, A. A. , and M. Fishbein . 2006. Plant defense syndromes. Ecology 87: S132‐S149. 16922309 10.1890/0012-9658(2006)87[132:pds]2.0.co;2

[ajb270176-bib-0004] Agrawal, A. A. , A. P. Hastings , M. T. J. Johnson , J. L. Maron , and J.‐P. Salminen . 2012. Insect herbivores drive real‐time ecological and evolutionary change in plant populations. Science 338: 113–116. 23042894 10.1126/science.1225977

[ajb270176-bib-0005] Arvanitis, L. , C. Wiklund , Z. Münzbergova , J. P. Dahlgren , and J. Ehrlén . 2010. Novel antagonistic interactions associated with plant polyploidization influence trait selection and habitat preference. Ecology Letters 13: 330–337. 20100239 10.1111/j.1461-0248.2009.01429.x

[ajb270176-bib-0006] Barco, B. , and N.K. Clay . 2019. Evolution of glucosinolate diversity via whole‐genome duplications, gene rearrangements, and substrate promiscuity. Annual Review of Plant Biology 70: 585–604. 10.1146/annurev-arplant-050718-100152 31035830

[ajb270176-bib-0007] Bates, D. , M. Maechler , B. Bolker , and S. Walker . 2015. Fitting linear mixed‐effects models using lme4. Journal of Statistical Software 67: 1‐48.

[ajb270176-bib-0008] Becerra, J.X. 2007. The impact of herbivore–plant coevolution on plant community structure. Proceedings of the National Academy of Sciences, USA 67: 7483–7488. 10.1073/pnas.0608253104 PMC1855276 17456606

[ajb270176-bib-0009] Bennett, M. D. 1987. Variation in genomic form in plants and its ecological implications. New Phytologist 106: 177–200.

[ajb270176-bib-0010] Biru, F. N. , T. Islam , X. Cibils‐Stewart , C. I. Cazzonelli , R. Elbaum , and S. N. Johnson . 2021. Anti‐herbivore silicon defences in a model grass are greatest under Miocene levels of atmospheric CO_2_ . Global Change Biology 27: 2959‐2969 33772982 10.1111/gcb.15619

[ajb270176-bib-1002] Boalt, E. , L. Arvanitis , K. Lehtilä , and J. Ehrlén . 2010. The association among herbivory tolerance, ploidy level, and herbivory pressure in Cardamine pratensis. Evolutionary Ecology 24: 1101–1113.

[ajb270176-bib-0011] Brosemann J , R. Overson , A.J. Cease , S. Millerwise , and M. Le Gall . 2023. Nutrient supply and accessibility in plants: effect of protein and carbohydrates on Australian plague locust (*Chortoicetes terminifera*) preference and performance. Frontiers in Insect Sciences 3: 1110518. 10.3389/finsc.2023.1110518 PMC10926423 38469479

[ajb270176-bib-0012] Catalán, P. , J. Müller , R. Hasterok , G. Jenkins , L. A. J. Mur , T. Langdon , A. Betekhtin , et al. 2012. Evolution and taxonomic split of the model grass *Brachypodium distachyon* . Annals of Botany 109: 385–405. 22213013 10.1093/aob/mcr294 PMC3268539

[ajb270176-bib-1003] Dinh Thi, V. H. , O. Coriton , I. Le Clainche , D. Arnaud , S. P. Gordon , G. Linc , P. Catalan , et al. 2016. Recreating Stable Brachypodium hybridum Allotetraploids by Uniting the Divergent Genomes of B. distachyon and B. stacei. PloS One 11: e0167171. 27936041 10.1371/journal.pone.0167171 PMC5147888

[ajb270176-bib-0013] Doyle, J. J. , and J. E. Coate . 2019. Polyploidy, the nucleotype, and novelty: The impact of genome doubling on the biology of the cell. International Journal of Plant Sciences 180: 1–52.

[ajb270176-bib-0014] Edger, P. P. , H. M. Heidel‐Fischer , M. Bekaert , J. Rota , G. Glöckner , A. E. Platts , D. G. Heckel , et al. 2015. The butterfly plant arms‐race escalated by gene and genome duplications. Proceedings of the National Academy of Sciences, USA 112: 8362–8366. 10.1073/pnas.1503926112 PMC4500235 26100883

[ajb270176-bib-0015] Fornoni, J. 2011. Ecological and evolutionary implications of plant tolerance to herbivory. Functional Ecology 25: 399‐407.

[ajb270176-bib-0016] Frew, A. , L. A. Weston , O. L. Reynolds , and G. M. Gurr . 2018. The role of silicon in plant biology: a paradigm shift in research approach. Annals of Botany 121: 1265–1273. 29438453 10.1093/aob/mcy009 PMC6007437

[ajb270176-bib-0017] Gaynor, M. L. , S. Lim‐Hing , and C. M. Mason . 2020. Impact of genome duplication on secondary metabolite composition in non‐cultivated species: a systematic meta‐analysis. Annals of Botany 126: 363–376. 32504537 10.1093/aob/mcaa107 PMC7424755

[ajb270176-bib-0018] Gordon, S. P. , B. Contreras‐Moreira , J. J. Levy , A. Djamei , A. Czedik‐Eysenberg , V. S. Tartaglio , A. Session , et al. 2020. Gradual polyploid genome evolution revealed by pan‐genomic analysis of *Brachypodium hybridum* and its diploid progenitors. Nature Communications 11: 3670. 10.1038/s41467-020-17302-5 PMC7391716 32728126

[ajb270176-bib-0019] Grinnan, R. , T. E. Carter Jr. , and M. T. J. Johnson . 2013. The effects of drought and herbivory on plant–herbivore interactions across 16 soybean genotypes in a field experiment: drought and plant–herbivore interactions. Ecological Entomology 38: 290–302.

[ajb270176-bib-0020] Hall, C. R. , V. Dagg , J. M. Waterman , and S. N. Johnson . 2020. Silicon alters leaf surface morphology and suppresses insect herbivory in a model grass species. Plants 9: 643. 32438683 10.3390/plants9050643 PMC7285219

[ajb270176-bib-0021] Hambäck, P. A. , and A. P. Beckerman . 2003. Herbivory and plant resource competition: a review of two interacting interactions. Oikos 101: 26–37.

[ajb270176-bib-0022] Hanley, M. E. , and R. J. Sykes . 2009. Impacts of seedling herbivory on plant competition and implications for species coexistence. Annals of Botany 103: 1347–1353. 19351683 10.1093/aob/mcp081 PMC2685311

[ajb270176-bib-0023] Harms, N. E. , and D. J. Walter . 2021. Influence of *Butomus umbellatus* L. lineage and age on leaf chemistry and performance of a generalist caterpillar. Aquatic Botany 172: 103391.

[ajb270176-bib-0024] Hull‐Sanders, H. M. , R. H. Johnson , H. A. Owen , and G. A. Meyer . 2009. Effects of polyploidy on secondary chemistry, physiology, and performance of native and invasive genotypes of *Solidago gigantea* (Asteraceae). American Journal of Botany 96: 762–770. 21628231 10.3732/ajb.0800200

[ajb270176-bib-0025] Ivey, C. T. , D. E. Carr , and M. D. Eubanks . 2009. Genetic variation and constraints on the evolution of defense against spittlebug (*Philaenus spumarius*) herbivory in *Mimulus guttatus* . Heredity 102: 303–311. 19092760 10.1038/hdy.2008.122

[ajb270176-bib-0026] Knight C. A. , N.A. Molinari , and D.A. Petrov 2005. The large genome constraint hypothesis: evolution, ecology and phenotype. Annals of Botany 95: 177‐190. 15596465 10.1093/aob/mci011 PMC4246716

[ajb270176-bib-1004] König, M. A. E. , K. Lehtilä , C. Wiklund , and J. Ehrlén . 2014. Among‐population variation in tolerance to larval herbivory by Anthocharis cardamines in the polyploid herb Cardamine pratensis. PloS One 9: e99333. 24945875 10.1371/journal.pone.0099333 PMC4063699

[ajb270176-bib-0027] Kuznetsova, A. , P. B. Brockhoff , and R. H. B. Christensen . 2017. lmerTest package: tests in linear mixed effects models. Journal of Statistical Software 82: 1‐26.

[ajb270176-bib-0028] Lenth, R. 2019. emmeans: Estimated marginal means, aka least‐squares means. R package version 1.3.4. Website https://CRAN.R-project.org/package=emmeans.

[ajb270176-bib-0029] López‐Alvarez, D. , A. J. Manzaneda , P. J. Rey , P. Giraldo , E. Benavente , J. Allainguillaume , L. Mur , et al. 2015. Environmental niche variation and evolutionary diversification of the *Brachypodium distachyon* grass complex species in their native circum‐Mediterranean range. American Journal of Botany 102: 1073–1088. 26199365 10.3732/ajb.1500128

[ajb270176-bib-0030] Lou, Y. , and I. T. Baldwin . 2003. *Manduca sexta* recognition and resistance among allopolyploid *Nicotiana* host plants. Proceedings of the National Academy of Sciences, USA 100: 14581–14586. 10.1073/pnas.2135348100 PMC304122 14530394

[ajb270176-bib-0031] Maddox, G. D. , and R. B. Root . 1987. Resistance to diverse species of herbivorous insects within a population of goldenrod, *Solidago altissima*: genetic variation and heritability. Oecologia 7: 2‐8–14. 10.1007/BF00385037 28312889

[ajb270176-bib-0032] Manzaneda, A. J. , K. V. S. K. Prasad , and T. Mitchell‐Olds . 2010. Variation and fitness costs for tolerance to different types of herbivore damage in *Boechera stricta* genotypes with contrasting glucosinolate structures. New Phytologist 188: 464–477. 20663059 10.1111/j.1469-8137.2010.03385.x PMC2950872

[ajb270176-bib-0033] Manzaneda, A. J. , P. J. Rey , J. T. Anderson , E. Raskin , C. Weiss‐Lehman , and T. Mitchell‐Olds . 2015. Natural variation, differentiation, and genetic trade‐offs of ecophysiological traits in response to water limitation in *Brachypodium distachyon* and its descendent allotetraploid *B. hybridum* (Poaceae). Evolution: 69: 2689–2704. 26377138 10.1111/evo.12776 PMC4605884

[ajb270176-bib-0034] Manzaneda, A. J. , P. J. Rey , J. M. Bastida , C. Weiss‐Lehman , E. Raskin , and T. Mitchell‐Olds . 2012. Environmental aridity is associated with cytotype segregation and polyploidy occurrence in *Brachypodium distachyon* (Poaceae). New Phytologist 193: 797–805. 22150799 10.1111/j.1469-8137.2011.03988.x PMC3257369

[ajb270176-bib-0035] Martínez, L. M. , A. Fernández‐Ocaña , P. J. Rey , T. Salido , F. Amil‐Ruiz , and A. J. Manzaneda . 2018. Variation in functional responses to water stress and differentiation between natural allopolyploid populations in the *Brachypodium distachyon* species complex. Annals of Botany 121: 1369–1382. 29893879 10.1093/aob/mcy037 PMC6007385

[ajb270176-bib-0036] Massey, F. P. , A. R. Ennos , and S. E. Hartley . 2006. Silica in grasses as a defence against insect herbivores: contrasting effects on folivores and a phloem feeder. Journal of Animal Ecology 75: 595–603. 16638012 10.1111/j.1365-2656.2006.01082.x

[ajb270176-bib-0037] Meunier, J. D. , D. Barboni , M. Anwar‐ul‐haq , P. Chaurand , V. Vidal , O. Grauby , R. Huc , et al. 2017. Effect of phytoliths for mitigating water stress in durum wheat. New Phytologist 215: 229–239. 28394079 10.1111/nph.14554

[ajb270176-bib-0038] Mitchell, C. , R. M. Brennan , J. Graham , and A. J. Karley . 2016. Plant defense against herbivorous pests: exploiting resistance and tolerance traits for sustainable crop protection. Frontiers in Plant Science 7: 1132. 27524994 10.3389/fpls.2016.01132 PMC4965446

[ajb270176-bib-0039] Núñez‐Farfán, J. , J. Fornoni , and P. L. Valverde . 2007. The evolution of resistance and tolerance to herbivores. Annual Review of Ecology, Evolution, and Systematics 38: 541–566.

[ajb270176-bib-0040] Pontes, L. D. A. S. , J.‐F. Soussana , F. Louault , D. Andueza , and P. Carrère . 2007. Leaf traits affect the above‐ground productivity and quality of pasture grasses. Functional Ecology 21: 844–853.

[ajb270176-bib-0041] R Core Team . 2019. R: a language and environment for statistical computing. R Foundation for Statistical Computing, Vienna, Austria. Website https://www.R-project.org/.

[ajb270176-bib-0042] Reese, A. T. , G. M. Ames , and J. P. Wright . 2016. Variation in plant response to herbivory underscored by functional traits. PLoS One 11: e0166714. 27936155 10.1371/journal.pone.0166714 PMC5147848

[ajb270176-bib-0043] Rey, P. J. , A. J. Manzaneda , and J. M. Alcántara . 2017. The interplay between aridity and competition determines colonization ability, exclusion and ecological segregation in the heteroploid *Brachypodium distachyon* species complex. New Phytologist 215: 85–96. 28436561 10.1111/nph.14574

[ajb270176-bib-0044] Sanchez, G. 2013. DiscriMiner: tools of the trade for discriminant analysis. R package version 0.1‐29. Website https://rdocumentation.org/packages/DiscriMiner/versions/0.1-29.

[ajb270176-bib-0045] Schädler, M. , G. Jung , H. Auge , and R. Brandl . 2003. Palatability, decomposition and insect herbivory: patterns in a successional old‐field plant community. Oikos 103: 121–132.

[ajb270176-bib-0046] Scholes, D. R. 2020. Ploidy in plant tolerance to apical meristem damage: A test of relative costs and benefits. International Journal of Plant Sciences 181: 509–517.

[ajb270176-bib-0047] Scholes, D. R. , and K. N. Paige . 2014. Plasticity in ploidy underlies plant fitness compensation to herbivore damage. Molecular Ecology 23: 4862–4870. 25145792 10.1111/mec.12894

[ajb270176-bib-0048] Scholthof, K.‐B. G. , S. Irigoyen , P. Catalan , and K. K. Mandadi . 2018. *Brachypodium*: a monocot grass model genus for plant biology. Plant Cell 30: 1673–1694. 29997238 10.1105/tpc.18.00083 PMC6139682

[ajb270176-bib-0049] Schranz, M. E. , A. J. Manzaneda , A. J. Windsor , M. J. Clauss , and T. Mitchell‐Olds . 2009. Ecological genomics of *Boechera stricta*: identification of a QTL controlling the allocation of methionine‐ vs branched‐chain amino acid‐derived glucosinolates and levels of insect herbivory. Heredity 102: 465–474. 19240753 10.1038/hdy.2009.12 PMC2775550

[ajb270176-bib-1005] Segraves, K. A. , and T. J. Anneberg . 2016. Species interactions and plant polyploidy. American Journal of Botany 103: 1326–1335. 27370313 10.3732/ajb.1500529

[ajb270176-bib-1006] Soltis, D. E. , C. J. Visger , D. B. Marchant , and P. S. Soltis . 2016. Polyploidy: Pitfalls and paths to a paradigm. American Journal of Botany 103: 1146–1166. 27234228 10.3732/ajb.1500501

[ajb270176-bib-0050] Stevens, M. T. , D. M. Waller , and R. L. Lindroth . 2007. Resistance and tolerance in *Populus tremuloides*: genetic variation, costs, and environmental dependency. Evolutionary Ecology 21: 829–847.

[ajb270176-bib-0051] Strauss, S. Y. , and A. A. Agrawal . 1999. The ecology and evolution of plant tolerance to herbivory. Trends in Ecology and Evolution 14: 179–185. 10322530 10.1016/s0169-5347(98)01576-6

[ajb270176-bib-0052] Strauss, S. Y. , J. A. Rudgers , J. A. Lau , and R. E. Irwin . 2002. Direct and ecological costs of resistance to herbivory. Trends in Ecology and Evolution 17: 278–285.

[ajb270176-bib-0053] Strauss, S. Y. , and A.R. Zangerl . 2002. Plant–insect interactions in terrestrial ecosystems. *In* C. M. Herrera and O. Pellmyr [eds.], Plant–animal interactions: an evolutionary approach, 77–106. Blackwell Science, Malden, MA, USA.

[ajb270176-bib-0054] Stutz, S. , H. L. Hinz , K. Konowalik , H. Müller‐Schärer , C. Oberprieler , and U. Schaffner . 2016. Ploidy level in the genus *Leucanthemum* correlates with resistance to a specialist herbivore. Ecosphere 7: e01460.

[ajb270176-bib-0055] te Beest, M. , J. J. Le Roux , D. M. Richardson , A. K. Brysting , J. Suda , M. Kubesová , and P. Pysek . 2012. The more the better? The role of polyploidy in facilitating plant invasions. Annals of Botany 109: 19–45. 22040744 10.1093/aob/mcr277 PMC3241594

[ajb270176-bib-0056] Vogel, J. P. , M. Tuna , H. Budak , N. Huo , Y. Q. Gu , and M. A. Steinwand . 2009. Development of SSR markers and analysis of diversity in Turkish populations of *Brachypodium distachyon* . BMC Plant Biology 9: 88. 19594938 10.1186/1471-2229-9-88 PMC2719641

[ajb270176-bib-0057] Wise, M. J. , and D. E. Carr . 2008. On quantifying tolerance of herbivory for comparative analyses. Evolution 62: 2429–2434. 18637836 10.1111/j.1558-5646.2008.00458.x

[ajb270176-bib-0058] Züst, T. , and A. A. Agrawal 2017. Trade‐offs between plant growth and defense against insect herbivory: an emerging mechanistic synthesis. Annual Review of Plant Biology 68: 513–534. 10.1146/annurev-arplant-042916-040856 28142282

