# Supplementary material for: Drivers of insect herbivory resistance and tolerance to plant damage in the Brachypodium distachyon species complex
Source: Am J Bot. 2026 Mar 16;113(4):e70176. doi: 10.1002/ajb2.70176 (PMC13103616; doi:10.1002/ajb2.70176)
Supplement: Supplementary file 1 — Appendix S1. Supplemental tables and figures. Figure S1: Details of the method followed to infest Brachypodium plants with locusts. Figure S2: Variation in plant damage (proportion of the available leaves consumed during bioassays) among populations of the Brachypodium distachyon species complex. Figure S3: Position of the three species of the Brachypodium distachyon species complex over the plane defined by the first two discriminant factors DF1 and DF2, obtained from discriminant analyses of four functional traits. Figure S4: Position of the B. hybridum populations and B. distachyon over the plane defined by the first two discriminant variables DF1 and DF2, obtained from discriminant analyses of four functional traits. Figure S5: Variation in functional traits silica content, C:N ratio, specific leaf area, and water content among B. hybridum and B. distachyon Iberian populations. Figure S6: Relationships between the C:N ratio and plant damage across B. distachyon genotypes. and relationships between silica content and plant damage across B. distachyon genotypes. Table S1: Brachypodium ID accessions and geographical origin of the plants included in the study. Table S2: Regression coefficients of the relationship between plant damage and fitness for each Brachypodium species. Table S3: Summary results of the general linear mixed model testing the effects of plant damage, population, and their interaction on three different maternal fitness components in Brachypodium distachyon. Table S4: Summary results of the general linear mixed model testing the effects of plant damage, population, and their interaction on three different maternal fitness components in Brachypodium hybridum. Table S5: Phenotypic correlations (Pearson's product–moment correlations) between leaf functional traits for Brachypodium distachyon and Brachypodium hybridum. Table S6: Phenotypic correlations (Pearson's product–moment correlations) between leaf functional traits for Brachypodium stacei. Tab [file AJB2-113-e70176-s001.docx]

**Appendix S1.** Supporting tables and figures.

**Table S1.** *Brachypodium* ID accessions and geographical origin of the plants included in the study.

| Species | Accession | Population | Coordinates | Annual  rainfall (mm) | Annual  temperature (°C) | Origin |
| --- | --- | --- | --- | --- | --- | --- |
| ***B. distachyon*** | Bd21 | Mosul | N/A | 622 | 14 | Iraq (USDA) |
|  | Bd30-1 | Sierra Nevada | 36º 59' 43'' N  3º 34' 18'' W | 440 | 10 | Spain (USDA) |
|  | SCA-5, SCA-7, SCA-8, SCA-9, SCA-10 | Sierra Nevada | 36º 59' 43'' N  3º 34' 18'' W | 440 | 10 | Spain (UJAEN) |
|  | CANU-4, CANU-13  CANU-14, CANU-15 | Algeciras | 36º 08' 37'' N  5º 30' 43'' W | 968 | 17 | Spain (UJAEN) |
|  | CER-2, CER-4, CER-5  CER-6, CER-16, CER-18 | Cervera | 42º 49' 32'' N  4º 29' 27'' W | 930 | 9 | Spain (UJAEN) |
|  | JHIN-10, JHIN-13 | Hinojares | 37º 43' 41'' N  2º 58' 52'' W | 725 | 11 | Spain (UJAEN) |
|  | VILLA-3, VILLA-4, VILLA-5, VILLA-6, VILLA-7, VILLA-13 | Villatoya | 39º 20' 32'' N  1º 20' 47" W | 397 | 16 | Spain (UJAEN) |
|  | RON-2, RON-3, RON-5  RON-7, RON-11 RON-13 | Roncal | 42º 46' 50" N  0º 57' 48'' W | 1102 | 11 | Spain (UJAEN) |
|  | GRAZ-1, GRAZ-5, GRAZ-7, GRAZ-10, GRAZ-16, GRAZ-20 | Grazalema | 36º 45' 21'' N  5º 26' 30'' W | 1773 | 16 | Spain (UJAEN) |
|  | CASIB-1, CASIB-8,  CASIB-10, CASIB-12,  CASIB-14, CASIB-15 | Casas Ibañez | 39º 16' 09'' N  1º 29' 22'' W | 409 | 13 | Spain (UJAEN) |
|  | SOBRE-2, SOBRE-6,  SOBRE-7, SOBRE-8,  SOBRE-18, SOBRE-19 | Sobredo | 42º 31' 54'' N  6º 51' 7'' W | 1008 | 12 | Spain (UJAEN) |
|  | VISC-4, VISC-8,  VISC-13, VISC-14 | Íscar | 41º 22' 12'' N  4º 32' 18'' W | 468 | 12 | Spain (UJAEN) |
| ***B. stacei*** | ALTAB-1, ALTAB-3,  ALTAB-5, ALTAB-12,  ALTAB-15 | Tabernas | 37º 02' 28'' N  2º 24' 22'' W | 298 | 16 | Spain (UJAEN) |
|  | CGAT-4, CGAT-6 | Cabo de Gata | 36º 43' 51'' N  2º 12' 4'' W | 311 | 19 | Spain (UJAEN) |
|  | ALSUR-1, ALSUR-2,  ALSUR-4 | Sorbas | 37º 05' 79'' N  2º 06' 35'' W | 342 | 18 | Spain (UJAEN) |
| ***B. hybridum*** | JHIN-8, JHIN-9, JHIN-11,  JHIN-16, JHIN-18, JHIN-19 | Hinojares | 37º 43' 41'' N  2º 58' 52'' W | 725 | 11 | Spain (UJAEN) |
|  | COPRE-4, COPRE-8, COPRE-9, COPRE-12, COPRE-13, COPRE-20 | Los Pedroches | 38º 36' 38'' N  5º 9' 3'' W | 542 | 16 | Spain (UJAEN) |
|  | FARO-1, FARO-2, FARO-3,  FARO-4, FARO-5, FARO-6 | Faro | 37º 0' 52'' N  7º 58' 35'' W | 776 | 16 | Portugal (UJAEN) |
|  | MON-1, MON-3, MON-10,  MON-13, MON-14 | Monfrague | 39º 49' 20'' N  6º 02' 52'' W | 807 | 18 | Spain (UJAEN) |
|  | RUI-1, RUI-9, RUI-10,  RUI-11 | Ruidera | 38º 57' 42 N  2º 52' 17" W | 433 | 14 | Spain (UJAEN) |
|  | JODAR-3, JODAR-6, JODAR-7, JODAR-8, JODAR-10, JODAR-19 | Jodar | 37º 48' 53'' N  3º 19' 09" W | 302 | 14 | Spain (UJAEN) |
|  | LARVA-1, LARVA-3, LARVA-8, LARVA-15,  LARVA-18, LARVA-20 | Larva | 37º 48' 38'' N  3º 12' 53" W | 500 | 14 | Spain (UJAEN) |
|  | MONE-2, MONE-3, MONE-4 MONE-5, MONE-6 | Monegros | 41º 30' 50'' N  0º 30' 30'' W | 349 | 15 | Spain (UJAEN) |
|  | ROS-3, ROS-6, ROS-7, ROS-9  ROS-10 | Rosas de Mar | 42º 14' 45'' N  3º 11' 1'' E | 591 | 14 | Spain (UJAEN) |
|  | JCIM-7, JCIM-9, JCIM-16  JCIM-18, JCIM-19, JCIM-20 | Cimbarra | 38º 23' 28'' N  3º 22' 13'' W | 509 | 14 | Spain (UJAEN) |

**Table S2**. Regression coefficients (±SE) of the relationship between plant damage and fitness (several fitness measurements considered) for each *Brachypodium* species. For negative coefficients, the steeper the slope, the less tolerant the plant is.

|  | Fitness metric | | |
| --- | --- | --- | --- |
| Species | Number of spikes | Number of seeds | Seed  biomass |
| *B. distachyon* | -0.15 (0.11) | 0.05 (0.16) | 0.003 (0.015) |
| *B. hybridum* | -0.16 (0.15) | -0.78 (0.2) | -0.07 (0.015) |
| *B. stacei* | -0.11 (0.31) | -0.74 (0.45) | -0.08 (0.04) |

**Table S3.** Summary results of the general linear mixed model testing the effects of plant damage, population, and their interaction on three different maternal fitness components in *Brachypodium* *distachyon*. A significant interaction of the plant damage x population term denotes inter-populational variation in tolerance to damage. Significant *P* values (<0.05) are in bold.

|  | Number  of spikes | | | | Seeds  (total number) | | | Seeds  (biomass) | | |
| --- | --- | --- | --- | --- | --- | --- | --- | --- | --- | --- |
| Effects | df | *F* | *P* | df | | *F* | *P* | df | *F* | *P* |
| Plant damage | 1,231 | 0.216 | 0.642 | 1,231 | | 0.19 | 0.672 | 1,230 | 0.001 | 0.973 |
| Population | 11,53 | 6.16 | **<0.0001** | 11,54 | | 7.44 | **<0.0001** | 11,53 | 5.29 | **<0.0001** |
| Plant damage x  Population | 11,236 | 1.84 | **0.047** | 11,237 | | 3.81 | **<0.0001** | 11,235 | 3.01 | **<0.0001** |
| Random effects | df | *LRT* | *P* | df | | *LRT* | *P* | df | *LRT* | *P* |
| Genotype (Population) | 1 | 10.47 | **0.0012** | 1 | | 7.04 | **0.0079** | 1 | 10.87 | **0.0009** |

**Table S4.** Summary results of the general linear mixed model testing the effects of plant damage, population, and their interaction on three maternal fitness components in *Brachypodium* *hybridum*. A significant interaction of the plant damage x population term denotes interpopulational variation in tolerance to damage. Significant P values (<0.05) are in bold.

|  | Number  of spikes | | | | Seeds  (total number) | | | Seeds  (biomass) | | |
| --- | --- | --- | --- | --- | --- | --- | --- | --- | --- | --- |
| Effects | df | *F* | *P* | df | | *F* | *P* | df | *F* | *P* |
| Plant damage | 1, 280 | 9.76 | **0.0019** | 1, 277 | | 28.85 | **<0.0001** | 1, 280 | 9.7 | **0.002** |
| Population | 9, 64 | 3.75 | **0.0008** | 9, 62 | | 1.74 | 0.097 | 9, 64 | 3.48 | **0.001** |
| Plant damage x  Population | 9, 279 | 1.73 | 0.081 | 9, 277 | | 0.92 | 0.51 | 9, 279 | 0.21 | 0.993 |
| Random effects | df | LRT | *P* | df | | LRT | *P* | df | LRT | *P* |
| Genotype (Population) | 1 | 15.91 | **<0.0001** | 1 | | 23.03 | **<0.0001** | 1 | 15.98 | **<0.0001** |

**Table S5**. Phenotypic correlations (Pearson's product–moment correlations) between leaf functional traits for *Brachypodium distachyon* (*N* = 41, above the diagonal) and *Brachypodium hybridum* (*N* = 56, below the diagonal). Significant correlations (*P* < 0.05) are in bold.

| Trait | Silica  content  (% DW) | C:N | Specific leaf area  (cm^2^ g^-1^) | Water content |
| --- | --- | --- | --- | --- |
| Silica content  (% dry mass) |  | **-0.646** | -0.002 | 0.078 |
| C:N | **-0.468** |  | -0.29 | -0.146 |
| Specific leaf area (cm^2^ g^-1^) | -0.13 | -0.03 |  | **0.497** |
| Water content | -0.208 | 0.158 | **0.3** |  |

**Table S6**. Phenotypic correlations (Pearson's product–moment correlations) between leaf functional traits for *Brachypodium stacei* (*N* = 9). Significant correlations (*P* < 0.05) are in bold.

| Trait | Silica content  (% DW) | C:N | Specific leaf area  (cm^2^ g^-1^) | Water content |
| --- | --- | --- | --- | --- |
| Silica content  (% DW) |  | 0.139 | 0.107 | -0.4 |
| C:N |  |  | -0.547 | 0.179 |
| Specific leaf area  (cm^2^ g^-1^) |  |  |  | **-**0.199 |
| Water content |  |  |  |  |

**Table S7**. Coefficients of the linear discriminant functions (DFs) of the four functional traits included in the discriminant analysis at the species level. Most important trait values in each discriminant variable are in bold.

| Trait | DF1 | DF2 |
| --- | --- | --- |
| Silica content  (% DW) | **-1.268** | **2.286** |
| C:N | 0.0737 | **0.725** |
| Specific leaf area  (cm^2^g^-1^) | -0.0038 | -0.029 |
| Water content | -0.1674 | -0.081 |

**Table S8**. Coefficients of the linear discriminant functions (DFs) of the four functional traits included in the discriminant analysis conducted at population level. The most important trait value in each discriminant variable is in bold.

| ***B. hybridum*** |  |  |
| --- | --- | --- |
| Trait | DF1 | DF2 |
| Silica content (% DW) | **-3.184** | **1.724** |
| C:N | -0.006 | 1.047 |
| Specific leaf area (cm^2^g^-1^) | 0.014 | 0.014 |
| Water content | 0.090 | -0.076 |
| ***B. distachyon*** |  |  |
| Trait | DF1 | DF2 |
| Silica content (% DW) | **1.962** | 0.247 |
| C:N | -0.081 | **-0.383** |
| Specific leaf area (cm^2^g^-1^) | -0.054 | 0.029 |
| Water content | 0.205 | -0.135 |


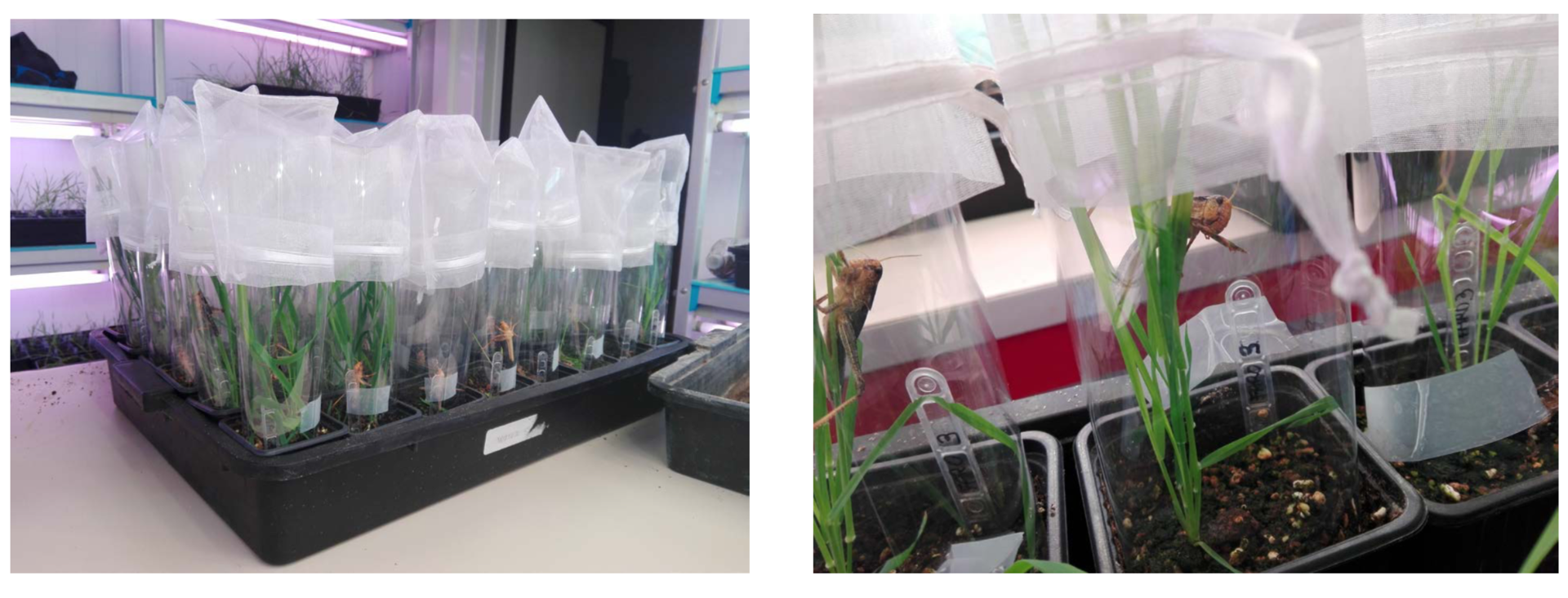


**Figure S1**. Details of the method to infest *Brachypodium* plants with locusts. Each plant–locust pair was enclosed in a cylindrical tube of acetate (3M) with is the top covered with an organza bag.

**
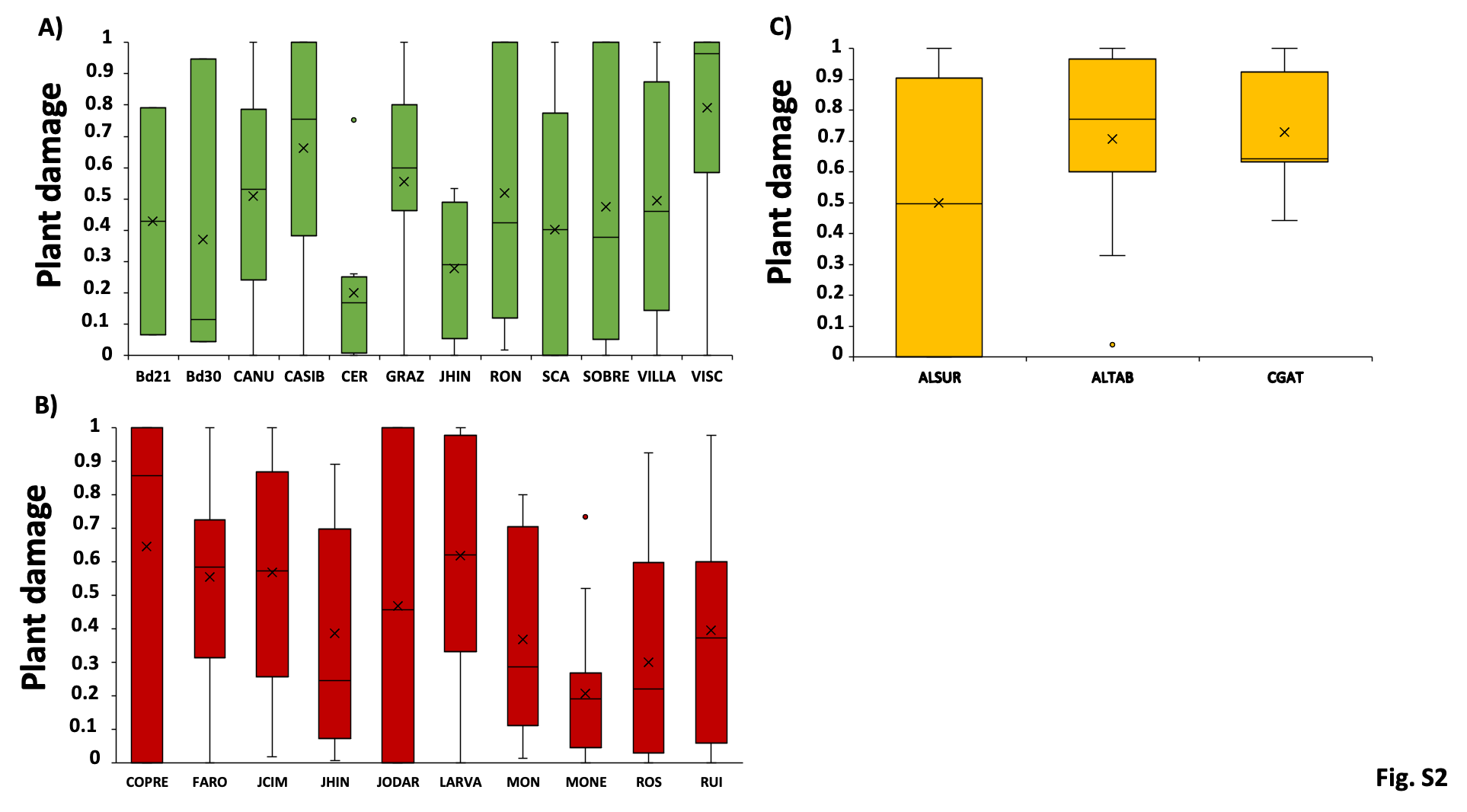
**

**Figure S2**. Variation in plant damage (proportion of available leaves consumed) among populations of the *Brachypodium* *distachyon* species complex. The box plots depict the whole variation of plant damage across (A) *B. distachyon* populations, (B) *B. hybridum*, and (C) *B. stacei*. Horizontal lines are the median; X depicts the estimated marginal mean.


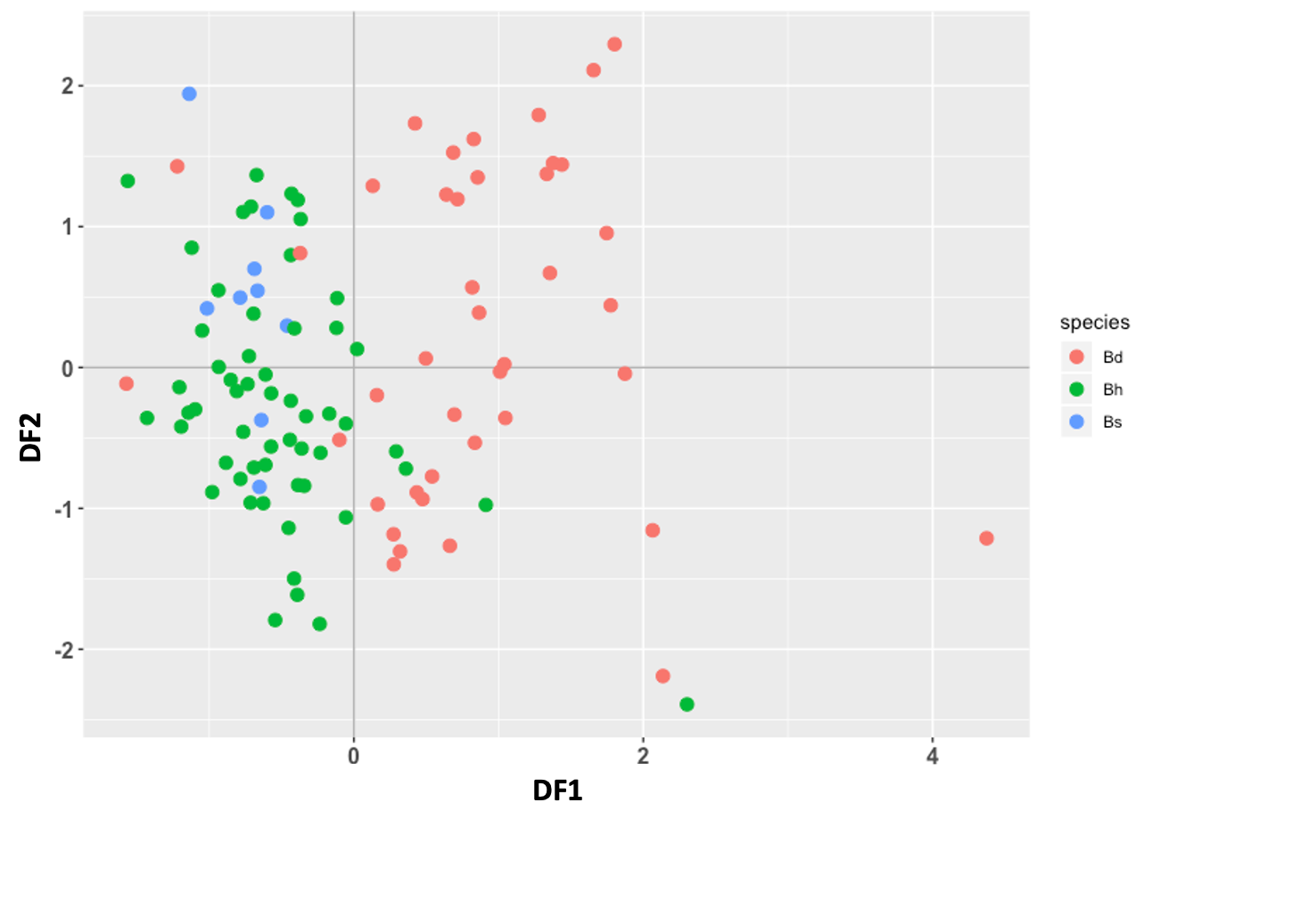


**Figure S3.** Position of the three species of the *Brachypodium distachyon* species complex over the plane defined by the first two discriminant factors DF1 and DF2 obtained from discriminant analyses of four functional traits. Bd, *B. distachyon*; (Bh) *B. hybridum*; Bs, *B. stacei*.


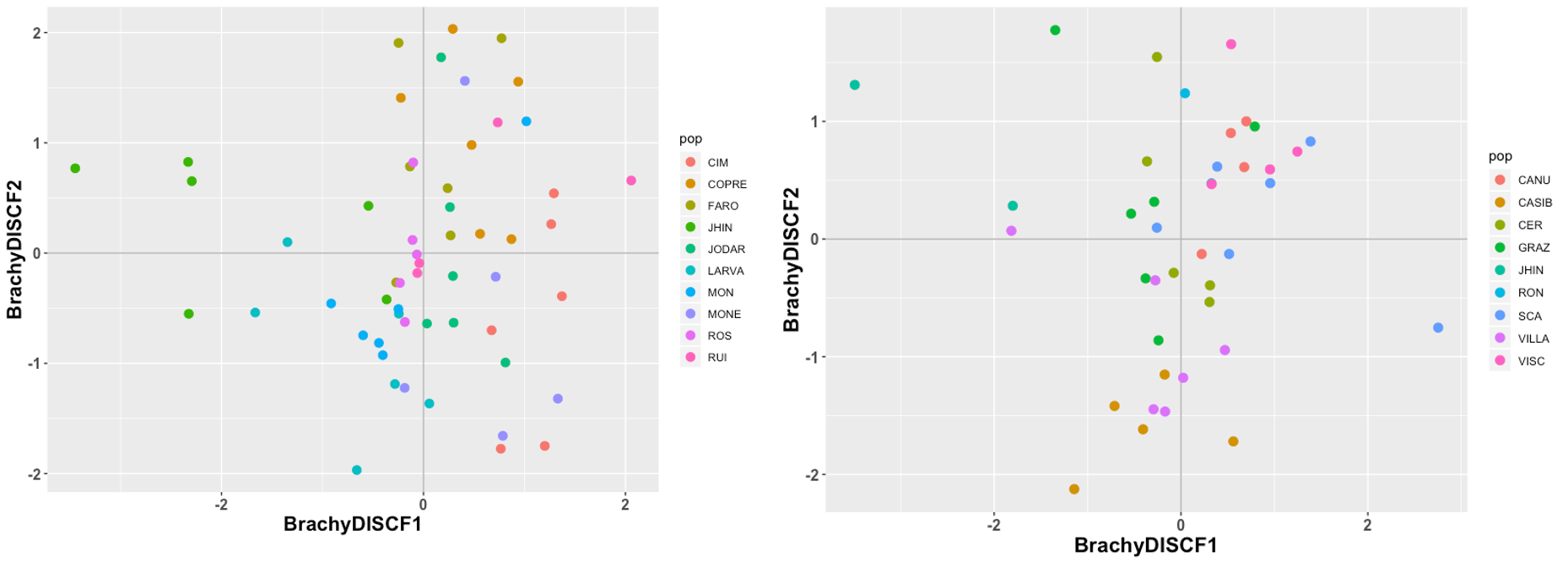


**Figure S4.** Position of *B. hybridum* populations (left) and *B. distachyon* (right) over the plane defined by the first two discriminant variables DF1 and DF2 obtained from discriminant analyses of four functional traits.


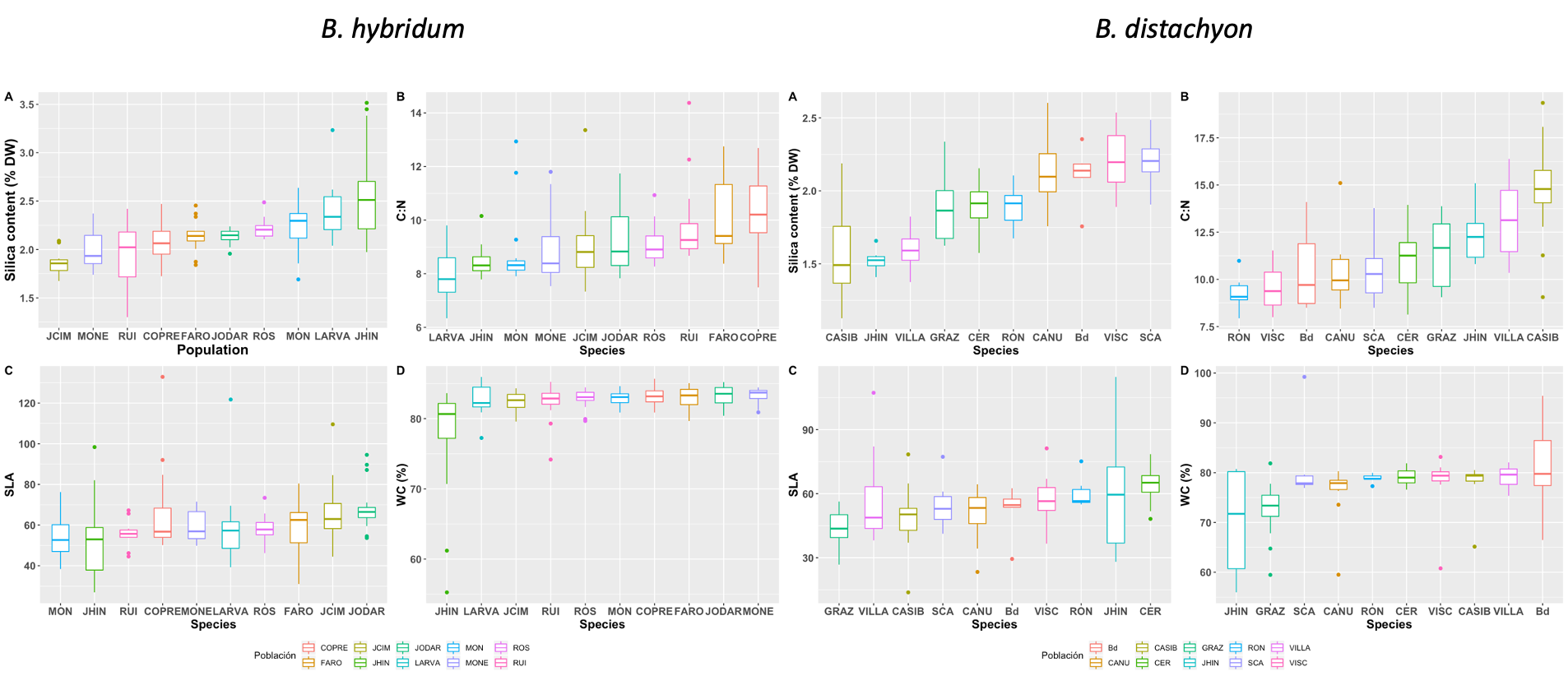


**Figure S5.** Variation in functional traits (A) silica content, (B) C:N ratio, (C) specific leaf area (SLA), and (D) water content (WC) between *B. hybridum* and *B. distachyon* Iberian populations.


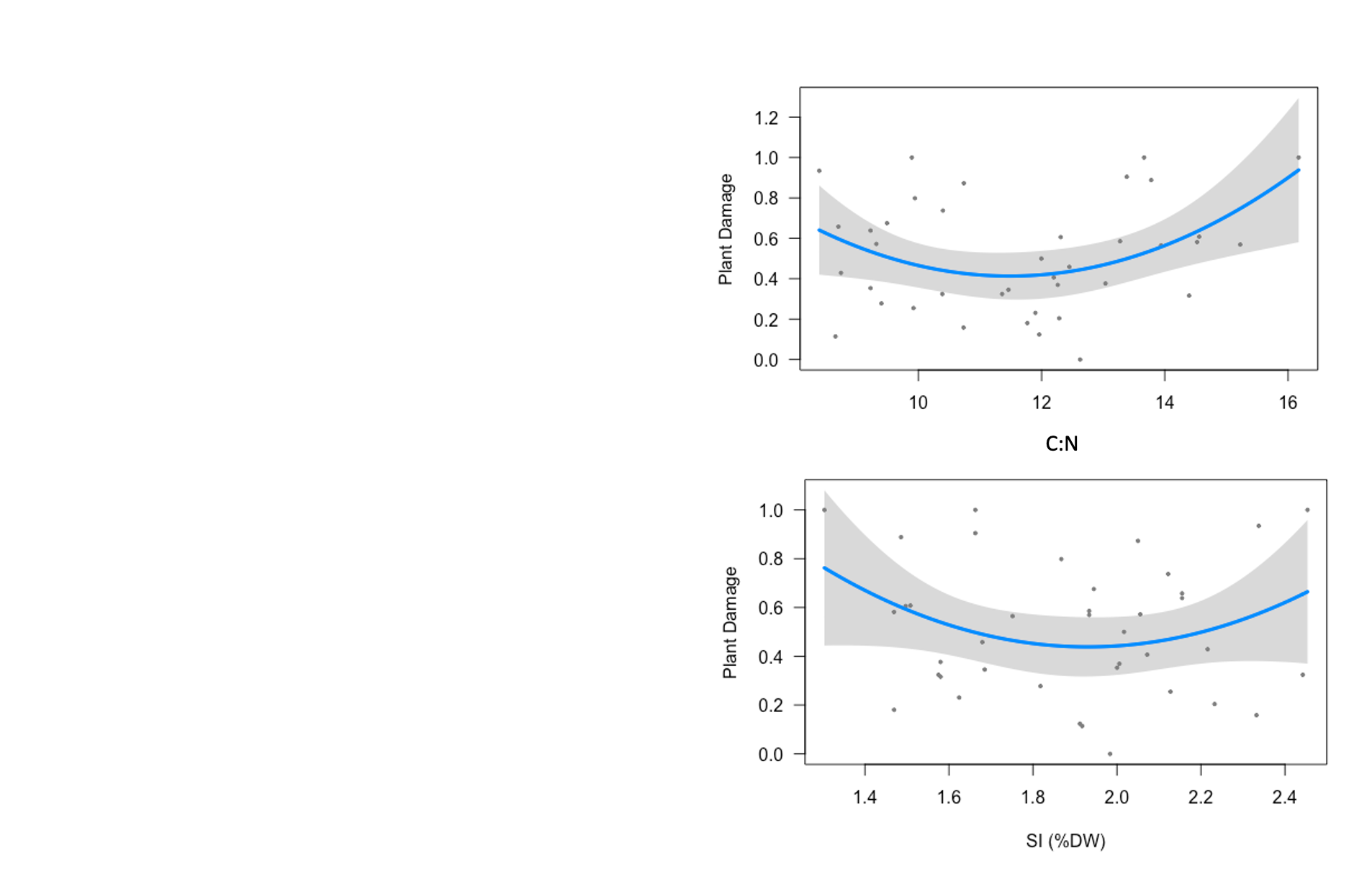


**Figure S6.** (Top) Relationship between C:N ratio and plant damage across *B. distachyon* genotypes. (Bottom) Relationship between silica content (SI, % of dry mass) and plant damage across *B. distachyon* genotypes. Solid lines depict quadratic regression fit between variables; dashed lines are 95% confidence intervals.
